# FlowClus: efficiently filtering and denoising pyrosequenced amplicons

**DOI:** 10.1186/s12859-015-0532-1

**Published:** 2015-03-27

**Authors:** John M Gaspar, W Kelley Thomas

**Affiliations:** Department of Molecular Cellular & Biomedical Sciences, University of New Hampshire, Durham, NH USA

**Keywords:** Metagenomics, Microbiome, Denoising, Pyrosequencing, 16S rRNA

## Abstract

**Background:**

Reducing the effects of sequencing errors and PCR artifacts has emerged as an essential component in amplicon-based metagenomic studies. Denoising algorithms have been designed that can reduce error rates in mock community data, but they change the sequence data in a manner that can be inconsistent with the process of removing errors in studies of real communities. In addition, they are limited by the size of the dataset and the sequencing technology used.

**Results:**

FlowClus uses a systematic approach to filter and denoise reads efficiently. When denoising real datasets, FlowClus provides feedback about the process that can be used as the basis to adjust the parameters of the algorithm to suit the particular dataset. When used to analyze a mock community dataset, FlowClus produced a lower error rate compared to other denoising algorithms, while retaining significantly more sequence information. Among its other attributes, FlowClus can analyze longer reads being generated from all stages of 454 sequencing technology, as well as from Ion Torrent. It has processed a large dataset of 2.2 million GS-FLX Titanium reads in twelve hours; using its more efficient (but less precise) trie analysis option, this time was further reduced, to seven minutes.

**Conclusions:**

Many of the amplicon-based metagenomics datasets generated over the last several years have been processed through a denoising pipeline that likely caused deleterious effects on the raw data. By using FlowClus, one can avoid such negative outcomes while maintaining control over the filtering and denoising processes. Because of its efficiency, FlowClus can be used to re-analyze multiple large datasets together, thereby leading to more standardized conclusions. FlowClus is freely available on GitHub (jsh58/FlowClus); it is written in C and supported on Linux.

**Electronic supplementary material:**

The online version of this article (doi:10.1186/s12859-015-0532-1) contains supplementary material, which is available to authorized users.

## Background

Amplicon-based metagenomics studies provide insight into the numbers and types of organisms in a particular sample based on DNA sequence analysis of a gene, such as the prokaryotic 16S ribosomal RNA gene [1]. The combined technologies of PCR and next-generation sequencing have allowed for the study of the rare biosphere by obviating the need for culturing or cloning. However, these same advances confound subsequent analysis of the sequence data. The presence of sequencing errors, PCR single-base errors, and PCR chimeras leads to inflated estimates of microbial diversity [2-7]. To limit the effects of these artifacts, various analytical tools have been designed. Two of the most widely used are AmpliconNoise [6] and the denoising pipeline in QIIME [3], the microbial ecology analysis package [8].

In Roche-454 pyrosequencing [9], as well as Ion Torrent sequencing [10], nucleotides are washed sequentially across a plate with picoliter-volume wells that each contain a bead attached to identical DNA molecules. If the flowed nucleotide is complementary to the templates in a well, a reaction occurs that results in the emission of light (or, with Ion Torrent, a change in pH) proportional to the number of nucleotides incorporated. This quantity of light is called a flow value. For example, if the nucleotide G is flowed and a well has a flow value of 4, it means that four Gs were added to the template. However, actual flow values are not integers; rather, they are floating-point values, such as 4.13. The flow values of a well over the course of a sequencing run, collectively referred to as the flowgram, are rounded to the nearest integers by the 454 software for sequence interpretation. If the 4.13 flow value were actually generated from a homopolymer of 5 Gs, then an incorrect deletion would have been made for that sequence. Hence, pyrosequencing errors, as well as errors in Ion Torrent sequencing [11], consist mostly of insertions and deletions, due to the incorrect determination of these nucleotide lengths, especially for longer homopolymers. Substitution errors are much rarer, since they occur only as the result of an overcall being followed by an undercall, or vice versa [9,12].

Reducing the effects of pyrosequencing errors is an important component in amplicon-based metagenomics, as well as in the many other applications that utilize such data [13-16]. In all these fields, various filtering criteria are used to remove entire sequence reads (elimination) or portions of reads (truncation) that are likely to contain many errors, while retaining as much sequence information as possible. Huse *et al.* [17], in analyzing first-generation (GS20) pyrosequencing, found that a relatively small number of reads accounted for the vast majority of the sequencing errors in their dataset. They concluded that removing reads that contained an ambiguous base (N) or that were of anomalous length was sufficient to achieve an error rate of 0.20% while retaining 93.3% of reads. In a similar study of second-generation (GS-FLX) pyrosequencing, Kunin *et al.* [4] used improved error estimates and an algorithm that took into account quality scores to approach the correct result for a known microbial sample. However, this was achieved at the expense of throwing out nearly 30% of the reads. With GS-FLX Titanium, seven variables contribute to the increased error rates of certain reads, including those relating to the location where a read is generated on the sequencing plate [18].

In amplicon-based metagenomics, there is no agreement on a set of filtering criteria that is universally used. Hence, the filtering step in QIIME allows the user to select from a variety of criteria based on sequences and quality scores. In AmpliconNoise, filtering is performed by analyzing flowgrams only, not sequences or quality scores. The filtering criteria are fixed and cannot be easily altered by the user. It has been shown that one particular criterion (truncating all flowgrams at the first flow value between 0.50 and 0.70), which remains unsubstantiated, is responsible for most of the eliminations and truncations of this step. In addition, although the filtering was designed to truncate reads prior to an ambiguous base, it misses most of these due to the way the flowgrams are analyzed [19].

With amplicon-based pyrosequences, there is an additional, fundamentally different approach for removing pyrosequencing errors known as denoising. In denoising, the reads are actually changed wherever a sequencing error is judged to have occurred. The principal insight behind an early denoising algorithm [2] was that the flowgrams contained information that could be used to aid in the identification of pyrosequencing errors. This algorithm’s successors, PyroNoise in AmpliconNoise and denoiser.py in QIIME, were similarly designed to cluster flowgrams, while reducing the prohibitive computational time of the original algorithm. Others have written software that further improved computational efficiency by disregarding flowgrams entirely [20].

Both PyroNoise and denoiser.py have user-selected parameters that control the denoising process. The default values for these parameters were chosen based on the analysis of mock community data, in which the correct sequences were known. Therefore, the parameters could be fine-tuned to minimize the error rates of reads. However, the algorithms were intended to be used in the studies of real communities. Without knowing the true sequences, one will not have a basis to choose values for the parameters other than the defaults.

We have previously shown that one can evaluate the outcomes of these denoising pipelines by considering how the individual reads have been changed at each step [19]. When used with the default parameter values, PyroNoise caused more substitutions than insertions or deletions, a pattern that is inconsistent with the known spectrum of pyrosequencing errors. By increasing one of the parameters (-s), we were able to achieve a result that was consistent with these errors. Another issue that arose was that of the “accordion effect”: since PyroNoise chose the longest read as the representative for each cluster, shorter reads had their 3’ ends filled in by sequences that were sometimes very different from what had been removed by truncation in the previous filtering step. The analysis was more complicated with denoiser.py in QIIME, since it also aligned the flowgrams during the denoising process, so as to allow for the correction of PCR single-base errors. Therefore, we could only point to the overall large number of changes (mostly substitutions), which were reduced with a change in the parameters.

In this paper, we detail our program, FlowClus, which both filters and denoises amplicon-based pyrosequenced data. Our goals for the program were fivefold: (1) to keep the users in charge of their data by allowing them to specify how the data are analyzed; (2) to provide feedback to users about how their data are being altered through the filtering and denoising processes, so they have a basis on which to adjust the parameters; (3) to avoid the negative side-effects of other denoising pipelines, such as the accordion effect; (4) to be able to analyze data generated by all stages of Roche-454 and Ion Torrent sequencing technology; (5) to require less computational resources than existing denoising algorithms, while still leveraging the information contained within the flowgrams.

## Methods

### Filtering

The different criteria by which certain reads are eliminated or truncated have a profound effect on the outcome from a denoising pipeline [19]. FlowClus allows one to choose from a number of criteria based on sequences, quality scores, and flowgrams, all of which are in the sff.txt file (produced by Roche-454 or Ion Torrent) that FlowClus requires as an input. The available criteria include those used by split_libraries.py in QIIME and CleanMinMax.pl in AmpliconNoise. The only default filtering is to require a match to a mid tag and primer, with the user being able to specify an allowed number of mismatches to each. Once a read matches a mid tag - primer, it is further analyzed according to the user-selected filtering criteria (see Additional file 1). When a read passes the filtering step, the flows corresponding to the mid tag and primer are removed from its flowgram, and the 3’ end of the flowgram is trimmed to match the read’s sequence, taking into account any truncations that were made.

At the end of the filtering step, FlowClus produces filtered flowgram files that are prepared for denoising, and a corresponding fasta file. It also creates a detailed report that lists the filtering criteria and the number of reads truncated and eliminated due to each, as well as the number of mid tag - primer matches and reads that passed the filtering for each sample. This report can be used to determine the effects of the filtering criteria on the data, and if, for example, a particular criterion has a biased effect on the reads derived from certain samples.

### Denoising

#### Clustering

To correct pyrosequencing errors, denoising algorithms such as the PyroNoise component of AmpliconNoise perform clustering of flowgrams. It is critical to understand what the meaning of such a clustering is. By clustering two flowgrams together, the algorithm is declaring that the two reads are so similar that the only differences between them are due to pyrosequencing errors. Therefore, it is acceptable to have them map to the same flowgram, even if that means changing one or both of them in some way to make them the same. Those changes are what the algorithm considers pyrosequencing errors.

The denoising part of FlowClus is based on similar logic. If two reads are so similar that the only differences between them are due to pyrosequencing errors, the two reads must have the same flow values at each position throughout their flowgrams, to within some margin of error. If any of the flow values differ by more than a given threshhold, then the reads probably contain differences beyond pyrosequencing errors, and they should not be clustered together. On the other hand, if each pair of flow values is not significantly different, then the reads are likely to be the same, except for pyrosequencing errors. Those reads can be clustered together and declared to be the same.

During denoising, FlowClus compares each read to the existing cluster centers in turn. If there are no significant differences between its flow values and that of a cluster center, it joins that cluster, and its flow values are averaged into the cluster center’s (based on a weighted average). If it does not match any of the existing clusters, it begins its own cluster.

Recalculating cluster centers in this manner can lead to drift, where early reads no longer match clusters whose centers have drifted away, and other reads that were not placed into clusters now are within the range. To address this issue, FlowClus iterates the process as follows. The first time through the reads, the cluster centers are created as described above. Next, the clusters centers are sorted based on the number of reads that map to them, from most populous to least. Then, the process begins again; each read is checked against the existing cluster centers. If its flow values are not significantly different from those of a cluster center, it joins that cluster, but the cluster center is not recalculated. Since the clusters are sorted by size after the first iteration, the reads will map to the most populous clusters possible.

At the end of the denoising process, FlowClus reconstitutes the reads by interpreting the clusters’ flowgrams. FlowClus rounds all flows ending in “.50” down, as the 454 software does most of the time, and adds an N whenever there are three consecutive flows of insufficient signal. The length of each read’s filtered flowgram is taken into consideration when its sequence is produced, so as to avoid the accordion effect [19]. In addition to this denoised fasta file, FlowClus produces output files that can be used for de novo PCR chimera checking by UCHIME [21] or Perseus [6].

#### Trie

An alternative usage for denoising with FlowClus is to utilize a trie data structure, in which the edges contain the flow values. Instead of comparing a query read to each of a set of clusters, the read is simply placed into the trie based on the same comparisons of flow values. As reads are placed, the flow values of the trie are updated, again based on a weighted average. In cases where a query flow value is within the denoising distance of multiple child edges, it is added to the closest edge.

Denoising with this method has greatly reduced computational requirements compared to the clustering approach, at the cost of some precision. In particular, there is no second iteration with the trie, making the denoising more sensitive to the choice of distance. Also, the abundance information provided for chimera checking is less apparent compared with clustering; the abundance of each leaf node is given by the number of reads that map to it or any of its ancestor nodes.

#### Denoising distance

The denoising process of FlowClus, whether by clustering or trie, is dependent on the choice of a distance threshhold, the maximum difference at which flow values are considered significantly different. This distance is determined by the user. One can choose a constant value, such as 0.50, which means that, for example, query flow values between 0.59 and 1.59 will not be considered significantly different from a reference flow value of 1.09. This is similar to how the 454 software interprets a flow value, except that 454 calls bases using integers as its reference values. Another possibility is to specify variable denoising distances. One can use distances based on the standard deviations of Balzer *et al.* [22], which increase with larger flow values and may better reflect 454 pyrosequencing errors. Or, one can use a set of custom distances that suit a particular dataset.

#### Outcome evaluation

The best way to evaluate the outcome of denoising is to ascertain whether or not the changes to the individual reads are consistent with the known spectrum of pyrosequencing errors. This is the same method we recommended for judging the outcomes of other denoising algorithms [19]. FlowClus provides an additional way for the user to assess the denoising process. As flow values are being compared, the program records these values whenever they are judged as being distinct, based on the user-specified denoising distance value. At the end of the denoising step, the user can visualize a levelplot of these “misses” to gain further insight as to whether the denoising value should be altered for a particular dataset.

### Datasets

For our primary analysis, we used a previously published dataset [19] derived from the microbiomes of fourteen individual nematodes. Two regions of the bacterial 16S ribosomal RNA gene (V3-V5 and V6-V8) were PCR amplified and sequenced using the Roche-454 GS FLX platform with the Titanium protocol (800 flows), resulting in just over 40,000 reads.

To calculate error rates, we retrieved the Titanium mock community dataset of Quince *et al.* [6], which was used to validate AmpliconNoise, as well as other denoising algorithms [23]. The 62,873 reads were derived from PCR amplification of the V4-V5 region of the 16S gene, using 91 plasmid clones as the source DNA (mock community). The set of original reads (“Stage 0”) was determined by filtering only for mid tag and primer sequences and allowing one and two mismatches to them, respectively. The initial error rate was calculated by finding the best match of each read to the 90 reference sequences (see Additional files 2 and 3) using ClustalW [24] with a reduced gap-opening penalty (-gapopen=1). In this and other error-rate calculations, we counted only insertions and deletions, which are the dominant form of errors in Roche-454 pyrosequencing [9]. We filtered the reads with FlowClus (version 1.1) using criteria similar to those recommended with the QIIME denoising pipeline and denoised with a constant value of 0.90. The dataset was also processed through the equivalent steps of AmpliconNoise V1.27 [6] and the denoising pipeline in QIIME 1.8.0 [8].

To evaluate scalability, we analyzed the large datasets from Krych *et al.* [25]. In this study of the human gut microbiome, the V3-V4 region of the 16S gene was amplified by PCR and sequenced on the Roche-454 GS FLX Titanium platform. The total number of reads for all three groups (baseline, synbiotic, and placebo) was 2.2 million.

## Results and discussion

We used FlowClus to filter and to denoise a previously published dataset [19].

### Filtering

With our dataset, 40,627 reads matched one of 56 different mid tag - primer combinations (14 samples, two amplicons, sequenced bidirectionally). We used several common filtering criteria, resulting in the elimination of 14.9% of the reads (Table 1). Most of these eliminations were due to reads that were shorter than the specified minimum sequence length of 200 bp, or that were truncated prior to this length by having a window of 50 quality scores whose average was less than 25. This sliding window criterion also resulted in the truncation of nearly half of the 34,592 reads that passed this step. The 12,486 reads that were truncated by removal of the reverse primer were almost exclusively from the shorter of the two amplicons, as would be expected.

**Table 1:** **Results of the filtering step of FlowClus**

	**Min. sequence length (200)**	**Max. sequence length for elimination (600)**	**Max. ambiguous bases allowed before truncation (0)**	**Reverse primer removed**	**Min. window quality score (length = 50, qual = 25.0)**	**Total**
Reads eliminated	2226	0	107	n/a	3702	6035
Reads truncated	n/a	n/a	1359	12486	15199	29044

### Denoising

#### Clustering

We denoised our dataset with FlowClus using a constant value of 0.50 for the maximum allowed difference between flow values. This resulted in 13,139 clusters of varying sizes. We determined what changes had been made at this step, using the same process we used to evaluate other denoising pipelines [19]. For the reference, we used the filtered reads whose flowgrams had been reinterpreted by FlowClus; these reads had 424 changes (mostly deletions) compared to the regular filtered reads, due to the rounding of flow values ending in “.50” down. The denoised reads had only 47 substitutions of the 4,225 total changes from the reference reads, with the remaining changes being slightly in favor of more deletions than insertions. This pattern is consistent with the known spectrum of pyrosequencing errors. In fact, of the 47 substitutions, 24 were conversions of an N (that had been interpreted by FlowClus after filtering) to a regular base.

**Figure 1:**
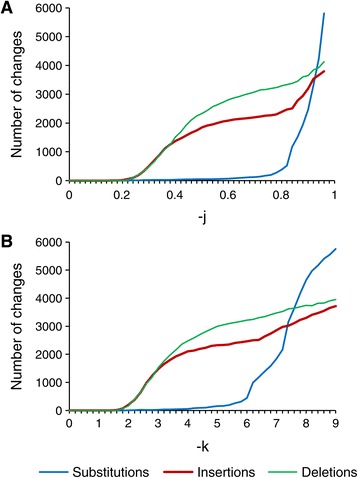
**Effects of altering the denoising distance parameter of FlowClus.** The numbers of changes to the reads made during denoising using different values for the maximum allowed distance between flow values. **A**: Effects of changing the constant denoising value (-j). **B**: Effects of using different multiples (-k) of the distances based on the standard deviations of Balzer *et al.* [22].

We further determined the effect of altering the constant value parameter of FlowClus. As this value (specified by the -j parameter) was increased, the numbers of insertions and deletions increased, as expected (Figure 1A). The number of substitutions remained much lower until the denoising value was increased above 0.80.

We also denoised using variable denoising distances based on the standard deviations of Balzer *et al.* [22]. When using different multiples of those distances (specified by the -k parameter), the changes were similar to those of specifying a constant denoising value, with the number of substitutions not increasing significantly until the multiple rose above six (Figure 1B).

**Figure 2:**
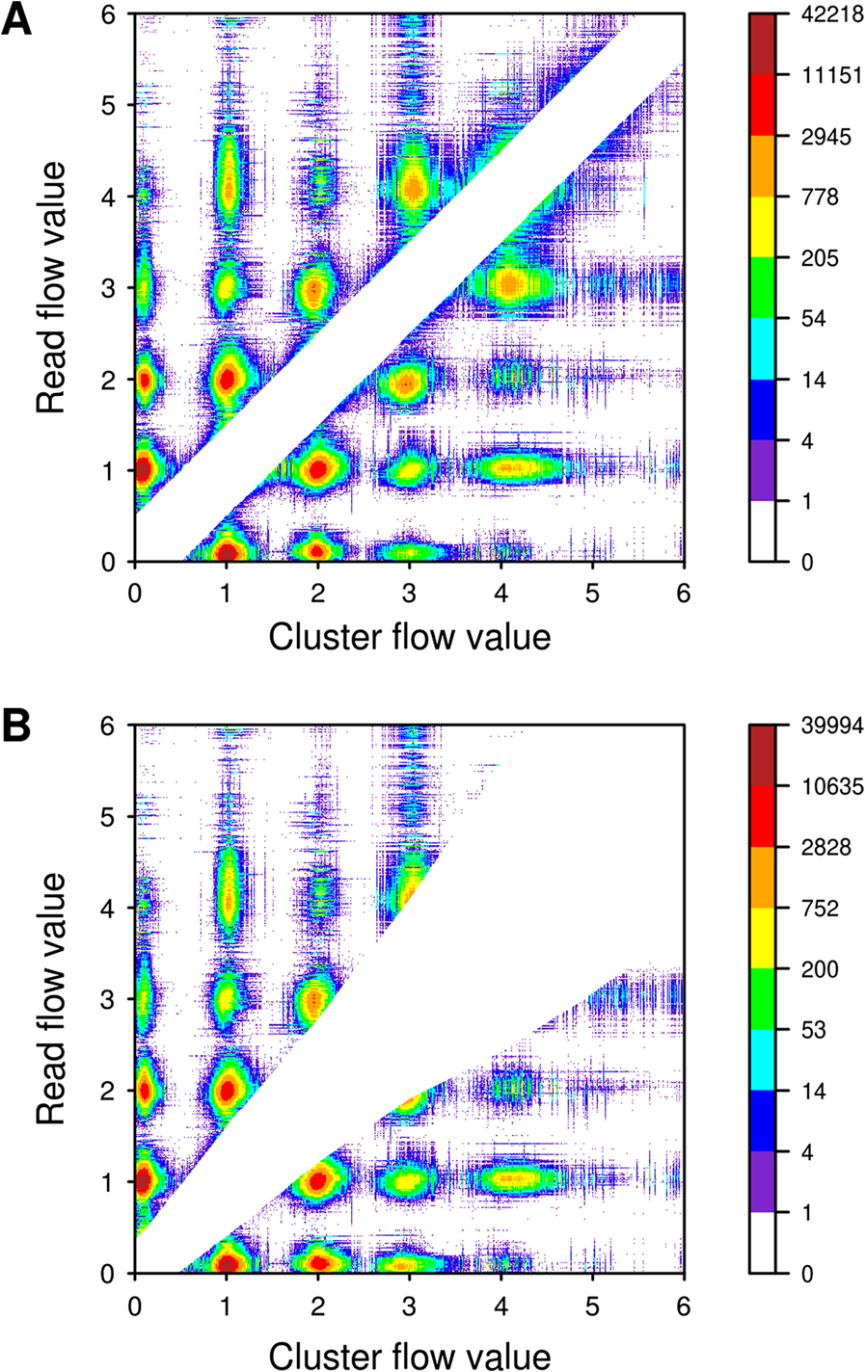
**Denoising “misses” with FlowClus.** As FlowClus denoises flowgrams by comparing pairs of flow values, it records the flow values of the cluster and the read each time they are judged as being distinct. The set of these “misses” for a denoising run can be visualized as a levelplot, such as those shown here. The red-orange colors represent a large number of misses at those particular pairs of cluster and read flow values. **A**: Denoising using a constant distance value of 0.50. **B**: Denoising using a multiple of five distances based on the standard deviations of Balzer *et al.* [22].

Although the best way to evaluate the outcome of denoising is to examine the changes to the reads, as we have done, FlowClus also allows one to visualize the set of flow values that were judged as being distinct during the denoising process. When we examined these denoising “misses” after using a constant distance value of 0.50, we saw an even white stripe that represented the flow values that were judged as not being different (Figure 2A). The denoising misses were concentrated around flow values close to integers, such as where the cluster had a flow value around one and the query read had a flow value close to zero. Between these local maxima and along the central stripe were blue and green “close” misses that suggested that using a larger constant denoising value might better suit this dataset. When denoising with a multiple of five for the distances of Balzer *et al.* [22], most of those close misses, especially at larger flow values, were not seen (Figure 2B).

#### Trie

With our dataset, the effects of altering the denoising values were similar to those of the clustering approach, except that the numbers of changes increased more sharply at larger distances (Additional file 4). Part of this volatility resulted from the trie analysis’ not including a second iteration through the reads.

After denoising with the trie and a constant value of 0.50, the misses did not have the clean white stripe down the middle that was seen previously (Additional file 5). This was due to cases where the query flow value was within the denoising distance of more than one child edge. A similar effect was seen when denoising with a multiple of five variable distances (Additional file 5).

### Benchmarking

#### Computational time

For our dataset, the filtering step of FlowClus required just over one minute (Table 2). This was 35 times faster than the filtering step in QIIME, and nearly 90 times faster than that of AmpliconNoise. Part of this discrepancy was explained by the fact that split_libraries.py (in QIIME) and SplitKeys.pl (in AmpliconNoise) needed to be run for each of the four primers for this dataset separately, whereas FlowClus analyzed them all at once.

**Table 2:** **Comparison of the run-times (in seconds) of different denoising pipelines**

	**FlowClus, min. filtering**	FlowClus, with filtering*	AmpliconNoise**	**QIIME**
Number of reads denoised	40627	34592	31868	36281
Filtering	63	64	5568	2211
Denoising	clustering: 16	clustering: 8	16536	35796
	trie: 3	trie: 2		

*Filtering options as shown in Table 1.

**Denoising by the PyroNoise step only.

When denoising by clustering with FlowClus, the run-time depends on the number of filtered reads, as well as the number of clusters formed. With our dataset that had been filtered, the denoising time was eight seconds, compared to 4.6 hours and 9.9 hours required by AmpliconNoise and QIIME, respectively (Table 2). FlowClus utilized a maximum of 64.4 MB memory, while PyroNoise and denoiser.py both needed more than twice that amount (145.5 MB and 235.9 MB, respectively).

It is important to note that AmpliconNoise, in addition to analyzing fewer reads due to its stringent filtering criteria, also denoised the reads by mid tag and primer separately (56 different bins for this dataset). QIIME and FlowClus both denoised by primer (four bins), which is a computationally more expensive approach, in time and memory usage. It is conceptually better to denoise data in as few groups as possible, allowing for error correction of reads derived from rare taxa [19].

With the dataset that had undergone minimal filtering, the denoising run-time of FlowClus doubled to 16 seconds. This was due to the increased number and lengths of reads, which in turn resulted in more clusters being formed. A maximum of 78.7 MB memory was required.

To assess scalability, we analyzed the “baseline” dataset of Krych *et al.* [25]. The 789,635 reads were filtered by FlowClus in two minutes, and denoising by clustering, with a constant distance of 0.50, required less than two hours and 2.6GB of memory (Additional file 6). AmpliconNoise, despite denoising 20.2% fewer reads (which were then binned by sample) and being parallelized over 16 cores, needed 2.6 days and 13.7GB of memory to process this dataset. The denoiser in QIIME, which was similarly run on 16 cores, took 7.9 days (Additional file 6), although running it on each sample separately (using the -S option) reduced the run-time to 1.4 days.

We further applied FlowClus to the combination of all three datasets (baseline, synbiotic, and placebo) of Krych *et al.* [25]. The 2.2 million raw reads were filtered in 5.5 minutes. Less than twelve hours and 7.1GB of memory were used to complete the denoising with a constant distance of 0.50 (Additional file 6).

When denoising by trie with FlowClus, the computational time becomes linear with respect to the number of reads. The time to denoise our filtered dataset was reduced to two seconds (Table 2), with only 8.5 MB of memory required. For the datasets of Krych *et al.* [25], the “baseline” reads were denoised in 36 seconds (423.6 MB memory), and the nearly 1.5 million filtered reads of the combined dataset were denoised in 97 seconds (1.2GB memory) (Additional file 6).

#### Error rates

In order to determine how effective a denoising algorithm has been in removing or correcting errors, one must analyze a dataset in which the correct sequences are known. To this end, datasets derived from mock communities are often used. Mock communities are created by combining known plasmid sequences or known bacterial genomic DNA. These DNA mixtures are then processed like a typical environmental sample, by PCR and sequencing.

**Figure 3:**
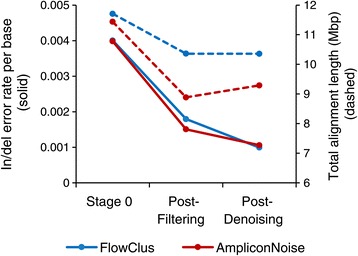
**Analyzing a mock community dataset.** A comparison of the error rates (solid lines) and total sequence alignment length (dashed lines) of the Titanium mock community dataset (Quince *et al.* [6]) analyzed by FlowClus and AmpliconNoise.

We examined the performance of FlowClus in correcting pyrosequencing errors in the Titanium mock community dataset of Quince *et al.* [6]. The set of original reads (“Stage 0”) was determined by filtering only for mid tag and primer sequences. The combined insertion and deletion error rate of these reads was just over 0.4% (Figure 3). We then filtered the reads with FlowClus using criteria similar to those recommended with the QIIME denoising pipeline. This resulted in a drop in the error rate by more than half, while losing 11.5% of the sequence information. We denoised the reads by clustering, using a constant 0.90 as the denoising distance, which was the largest value that did not cause a significant (>5%) change in the substitution error rate. After denoising, the in/del error rate was further reduced, to less than 0.1%.

We processed the mock community dataset through the equivalent steps of AmpliconNoise. The initial error rate was nearly identical to that of FlowClus, although there were fewer sequences, since SplitKeys.pl requires an exact match of the mid tag and primer [19]. After filtering with CleanMinMax.pl, the error rate was reduced further than with FlowClus, but this was achieved at the expense of losing nearly twice as much sequence information (Figure 3). The PyroNoise step of AmpliconNoise, which is designed to correct only pyrosequencing errors, brought the error rate down nearly to the level of FlowClus. However, it is important to note that this error rate was artificially deflated, because of the positive 3’ gap of PyroNoise [19], as shown by the increase in sequence information (Figure 3).

We also analyzed this mock community dataset with the QIIME denoising pipeline, and we found that the error rates through each step were similar to those of FlowClus (Additional file 7). Here again, though, the final error rate of just over 0.1% was artificially reduced because of the positive 3’ gap of denoiser.py. The lowest error rate was produced after filtering using the -g option in QIIME, but this also resulted in the loss of 25.6% more sequence information (Additional file 7).

In all these analyses, factors other than sequencing errors also contributed to the in/del error rate. These included in/dels derived from PCR errors and the possibility of some incorrect reference Sanger sequences (Additional file 2). In addition, the presence of reads derived from contaminants [23] and PCR chimeras also increased the error rates. We found that removing reads classified as chimeras by UCHIME [21] had a small and equivalent effect on the in/del error rates for each of the denoising pipelines. Despite these issues, we performed no manipulation or exclusion of individual reads, beyond what was done by the denoising algorithms.

In analyzing this dataset, FlowClus had a greatly decreased run-time compared to the other pipelines (Additional file 6). It filtered and denoised in just over twenty seconds. AmpliconNoise required more than nine hours, despite analyzing 13.4% fewer reads. The QIIME denoising pipeline used nearly seven hours, although some of this time was used to correct PCR single-base errors, which denoiser.py does simultaneously with addressing pyrosequencing errors.

When used with the trie denoising option, FlowClus required just two seconds (Additional file 6). The error rates across constant denoising distances ranging from 0.3 to 0.8 were similar to those when denoising by clustering (Additional file 8). However, the trie error rates greatly increased at denoising distances above 0.8. This underscores the sensitivity of the trie denoising option to larger denoising distances, which is consistent with the spectrum of read changes seen previously (Additional file 4).

## Conclusions

The importance of accounting for errors stemming from PCR and sequencing in amplicon-based metagenomic studies is well-established [2-7]. However, existing denoising programs have negative side-effects [19] and do not allow one to evaluate the outcome when they are used to analyze real-world data. In addition, they are computationally prohibitive for many larger datasets.

We have described a new program, FlowClus, that filters and denoises pyrosequenced amplicons. Our goal was to have a program that would keep the users in charge of their data by providing detailed information about the filtering and denoising processes, and that would be practical for use with current and future datasets, including those generated by Ion Torrent sequencing.

For filtering, FlowClus provides a wide variety of criteria that will eliminate or truncate reads based on sequences, quality scores, and flowgrams. The user can select any or all of these criteria, and the program analyzes the reads according to a strict order of operations. When the filtering step is completed, FlowClus provides an accounting of the effects that the chosen criteria have. If a particular criterion has a biased effect on certain samples, for example, the user will be made aware of this and may consider altering the filtering parameters accordingly. Although this step was also designed to prepare the flowgrams for denoising, those analyzing other pyrosequenced datasets may choose to filter their data using FlowClus, simply because of the value of the information that is reported back and the systematic approach that is employed.

The denoising process in FlowClus is designed to correct pyrosequencing errors. Like other denoising programs, it does this by clustering flowgrams whose only differences are judged as being pyrosequencing errors. With FlowClus, the user controls the clustering process by setting the maximum distance allowed between flow values. After the denoising has completed, the effects of denoising can be assessed by ascertaining whether or not the changes to the individual reads are consistent with the known spectrum of pyrosequencing errors. This is the same method we recommended for judging the outcomes of other denoising pipelines [19]. An additional way one can evaluate denoising with FlowClus is to examine a levelplot of the flow values that were considered different during the clustering process. Both of these methods provide for the user information that can be used to adjust the parameters to suit the particular dataset being analyzed.

The feedback provided for the filtering and denoising steps of FlowClus applies to datasets generated from real-world samples. That is, the user does not need to know the true sequences to judge the effects that the program has had on the data. This is an important distinction, because other denoising algorithms were validated based on their abilities to achieve the “correct” results with mock community data. The problem is that these results are not necessarily reflective of how well the algorithms will perform with data derived from real communities. In a real-world sample, there are rare variants of more dominant species, and it is not certain that these true sequences will not be considered errors. It is also unclear how well a denoising algorithm that was validated with mock community data at one stage of sequencing technology will continue to perform as the technology improves, generating more and longer reads per run.

Nevertheless, we processed a mock community dataset through FlowClus. It produced a lower error rate than AmpliconNoise and the QIIME denoising pipeline, while retaining more sequence information. Another important result from this analysis was the extent to which filtering reduced the error rates far more than denoising in each of the pipelines. This highlights the importance of continued study of filtering criteria, which does not seem to be keeping pace with new advances in sequencing technology.

FlowClus does not address sources of error arising from PCR artifacts directly. There are already numerous programs available that are used to identify PCR chimeras. Due to the importance of removing chimeras in amplicon-based metagenomics, we designed FlowClus to produce files that can be fed to de novo chimera-checking programs, which require abundance information (reference-based programs can be used in any case). On the other hand, we do not believe that PCR single-base errors contribute substantially to noise, nor do we believe there is a valid model that can distinguish such errors from the natural diversity inherent in real-world samples [19].

With all the datasets, FlowClus completed the analysis in a fraction of the time required by AmpliconNoise and the QIIME denoising pipeline. Our small dataset was processed in under two minutes. Less than twelve hours were required for a large dataset of 2.2 million reads. Those who are analyzing such large datasets can consider using FlowClus with the trie denoising option, which is even more efficient, at the cost of some precision. There is no second iteration with the trie, making the denoising more sensitive to the choice of distance, and the abundance information provided for chimera checking is less precise compared to that from clustering.

As sequencing technology has progressed, reads have increased in length and in quantity. FlowClus was not written for a particular implementation of Roche-454 sequencing, so it can analyze reads generated from any number of flows and any flow order (including flow pattern B). With the impending demise of pyrosequencing, Ion Torrent has gained popularity in the field; FlowClus can process data from this sequencing platform as well. This is particularly important in addressing the need to re-analyze multiple large datasets that may have been generated at different stages of sequencing technology. Because of its flexibility and efficiency, FlowClus is uniquely suited to be able to perform this task.

## Availability of supporting data

The dataset for our primary analysis, taken from Gaspar and Thomas [19], is available in the NCBI Sequence Read Archive, accession SRR653182. The Titanium mock community dataset of Quince *et al.* [6] is available in the SRA, accession SRR068370. The datasets of Krych *et al.* [25] are available in the SRA, accessions SRR550157-68.

## Additional files

Additional file 1:
**The filtering criteria and order of operations used by FlowClus.**
Additional file 2:
**Notes on the mock community dataset that was used.**
Additional file 3:
**The 90 reference sequences of the mock community dataset.**
Additional file 4:
**Effects of altering the parameters of FlowClus when using a trie.** The numbers of changes to the reads made during denoising with a trie was determined. A: Effects of changing the constant denoising value. B: Effects of using different multiples for the distances based on the standard deviations of Balzer *et al.* [22]. Additional file 5:
**Denoising “misses” with FlowClus when using a trie.** Levelplots of the flow values that were judged as being distinct, based on the following user-selected parameters. A: A constant value of 0.50. B: A multiple of five distances based on the standard deviations of Balzer *et al.* [22]. Additional file 6:
**Comparisons of the run-times (in seconds) of different denoising pipelines.** A: Titanium mock community dataset of Quince *et al.* [6]. B: Baseline dataset of Krych *et al.* [25]. C: Combined dataset (baseline, synbiotic, and placebo) of Krych *et al.* [25]. Additional file 7:
**Analyzing a mock community dataset by the QIIME denoising pipeline.** A comparison of the error rates (solid lines) and total sequence alignment length (dashed lines) of the Titanium mock community dataset [6] analyzed by the QIIME denoising pipeline, with and without the -g filtering option. Additional file 8:
**Comparisons of the error rates when denoising by clustering vs. by trie at different denoising distances.**

## Abbreviations

PCR: Polymerase chain reaction
