# Supplementary material for: FlowClus: efficiently filtering and denoising pyrosequenced amplicons
Source: BMC Bioinformatics. 2015 Mar 27;16(1):105. doi: 10.1186/s12859-015-0532-1 (PMC4380255; doi:10.1186/s12859-015-0532-1)
Supplement: Additional file 1: — The filtering criteria and order of operations used by FlowClus. [file 12859_2015_532_MOESM1_ESM.pdf]

Filtering order of operations

Sequences

- Minimum sequence length
- Maximum sequence length
- Maximum sequence length for truncation
- Maximum ambiguous bases
- Maximum ambiguous bases for truncation
- Maximum homopolymer length
- Maximum homopolymer length for truncation
- Remove reverse primer
- Require reverse primer (and remove)

Quality scores

- Average quality score
- Sliding window of quality scores (option to eliminate)

Flowgrams

- Minimum flowgram length
- Maximum flowgram length (for truncation)
- Noisy interval of flow values
- Maximum flow value
- No signal for 4 flows

During filtering by FlowClus, a given read is either eliminated, truncated, or neither. If multiple criteria would have eliminated or truncated a read, the criterion credited is the first in the categories listed above (Sequences, Quality Scores, Flowgrams). In analyzing the sequence or flowgram of a read, the read must first pass any length restrictions. After this, the sequence or flowgram is examined 5′ to 3′, and

the criterion that is violated first is credited with the elimination or truncation.

If a user selects the “Maximum ambiguous bases” criterion with a value of 2, any read that contains more than 2 ambiguous bases (Ns) will be eliminated. If the user selects the similar “Maximum ambiguous bases for truncation” criterion with a value of 2, a read will be truncated immediately prior to the third N in its sequence. This may still result in the read’s elimination, if the resulting sequence falls below the minimum specified by the “Minimum sequence length” or “Minimum flowgram length” criteria.

A complete description of these criteria, and the other parameters of FlowClus, is given in the README that accompanies the program.
