# Supplementary material for: FlowClus: efficiently filtering and denoising pyrosequenced amplicons
Source: BMC Bioinformatics. 2015 Mar 27;16(1):105. doi: 10.1186/s12859-015-0532-1 (PMC4380255; doi:10.1186/s12859-015-0532-1)
Supplement: Additional file 2: — Notes on the mock community dataset that was used. [file 12859_2015_532_MOESM2_ESM.pdf]

Mock community

The Titanium mock community data of Quince *et al.* [6] has been used to validate AmpliconNoise and other denoising algorithms [23]. The community was derived from a mixture of 91 plasmid clones. Eighty-eight of the sequences are available from GenBank (HQ462473-560). The reference sequences, attained through Sanger sequencing of the plasmids, contain more than 200 ambiguous bases (Ns). Further examination of these references revealed that eight are identical to other sequences (ignoring Ns), and that four differ by only one or two base-pairs from another reference.

These characteristics complicate the calculation of error rates. One cannot accurately determine the number of errors in a read that was derived from a reference whose sequence is unknown. It is also difficult to quantify the results for a read that is a close match to multiple references, or whose reference contains numerous Ns.

To ensure that we could more accurately map the sequence reads to the correct references, we performed the following procedure. For the reference database, we retrieved the 88 reference sequences from GenBank, and trimmed them with the primers used for amplification. We then reverse-complemented them, producing a total of 176 references. We filtered the raw reads with FlowClus, selecting only reads that were at least 200 bp long and had no more than one mismatch to the mid tag and two mismatches to the forward or reverse primer (the amplicons were sequenced bidirectionally). We then removed the reads that were determined by UCHIME [21] as

chimeras, using the 176 references as the database.

Using QIIME [8], we clustered the reads at 99% with uclust and picked a representative read for each cluster. Then we determined the closest reference for each of the representative reads by minimizing the error rate calculated after pairwise alignment to each of the 176 references with ClustalW [24].

For the reference sequences that had at least one N, we examined the most populous clusters that mapped to them. We found that many of the reference Ns were false insertions, with less than 10% of the reads in the cluster having bases at those positions. We deleted those Ns from the references. It has been asserted that other errors in the reference Sanger sequences exist [23], but we did not perform any additional manipulation of the references.

Next, we searched for the missing references. We found two representative reads from large clusters (at least 100 reads) that were poor matches to their closest references. The other reads in those clusters were independently determined to match the same references, but just as poorly as the representatives. Furthermore, the two references each had more populous clusters that mapped to them more closely. This suggested that the two clusters were derived from missing references, or that they were actually chimeras. To test the latter possibility, we analyzed the representative reads with both UCHIME and Perseus [6], using the 176 reference sequences as the database. When both were cleared as not being chimeras, we added them (454 accession numbers

GDUPB3C07IVPKT and GDUPB3C07IWQYP) to the references.

Our updated reference database used to calculate error rates consisted of 90 sequences -- the original 88 references from GenBank, trimmed using the primers, plus the two added sequences. Altogether, they contained 27 Ns.

The sequences can be found in Additional file 3.
