# Supplementary material for: FlowClus: efficiently filtering and denoising pyrosequenced amplicons
Source: BMC Bioinformatics. 2015 Mar 27;16(1):105. doi: 10.1186/s12859-015-0532-1 (PMC4380255; doi:10.1186/s12859-015-0532-1)
Supplement: Additional file 3: — The 90 reference sequences of the mock community dataset. [file 12859_2015_532_MOESM3_ESM.pdf]

>HQ462473
TACGGGGGGTGCAAGCGTTGTTCGGAATTATTGGGCGTAAAGCGCGTGTAGGCGGTTTGTTAAGTCTGTTGTG
AAAGCCCTGGGCTCAACCCAGGAAGTGCACTGGATACTGGCAGACTTGAATACGGGAGAGGGTAGTGGAATTC
CTGGTGTAGGAGTGAAATCCGTAGATATCAGGAGGAACACCGGTGGCGAAGGCGGCTACCTGGACCGATATTG
ACGCTGAGACGCGAAAGCGTGGGTAGCAAACAGGATTAGATACCCTGGTAGTCCACGCCGTAAACGATGAGTA
CTAGGTGTTGCGGGTATTGACCCCTGCAGTGCCGCAGCTAACGCATTAAGTACTCCGCCTGGGAAGTACGGTC
GCAAGACTA
>HQ462474
TACGTAGGGGCCAAGCGTTGTCCGGATTTATTGGGCGTAAAGAGCTCGTAGGCGGTTTGGATAGTCGGGTGTG
AAAACTCCGAGCTCAACTCGGAGACGCCACCCGATACTGCCATGACTAGAGTCCGGTAGGGGAGCAGGGAATT
CCTGGTGTAGCGGTGAAATGCGCAGATATCAGGAGGAACACCGGTGGCGAAGGCGCTGCTCTGGGCCGGTACT
GACGCTGAGGAGCGAAAGCGTGGGTAGCAAACAGGATTAGATACCCTGGTAGTCCACGCCGTAAACGTTGGGC
ACTAGGTGTGGGCCCTAACCAACGGGGTCCGTGCCGCAGCTAACGCATTAAGTGCCCCGCCTGGGGAGTACGG
CCGCAAGGCTA
>HQ462475
TACGAAGGGGGCTAGCGTTGCTCGGAATCACTGGGCGTAAAGCGCACGTAGGCGGACTCTTAAGTCGGGGGTG
AAATCNTGGAGCTCAACTCCAGAACTGCCTTCGATACTGAGAGTCTTGAGTCCGGGAGAGGTGAGTGGAACTG
CGAGTGTAGAGGTGAAATTCGTAGATATTCGCAAGAACACCAGTGGCGAAGGCGGCTCACTGGCCCGGTACTG
ACGCTGAGGTGCGAAAGCGTGGGGAGCAAACAGGATTAGATACCCTGGTAGTCCACGCCGTAAACGATGGATG
CTAGCCGTTGGCAGGCTTGCCTGTCAGTGGCGCAGCTAACGCATTAAGCATCCCGCCTGGGGAGTACGGTCGC
AAGATTA
>HQ462476
TACGTAGGGTGCAAGCGTTGTCCGGAATTATTGGGCGTAAAGAGCTCGTAGGCGGTCTGTCGCGTCGAATGTG
AAAACCCGGGGCTCAACTCCGGGCCTGCATTCGATACGGGCAGACTAGAGTTCGGTAGGGGAGACTGGAATTC
CTGGTGTAGCGGTGAAATGCGCAGATATCAGGAGGAACACCGGTGGCGAAGGCGGGTCTCTGGGCCGATACTG
ACGCTGAGGAGCGAAAGCGTGGGGAGCAAACAGGATTAGATACCCTGGTAGTCCACGCCGTAAACGTTGGGCG
CTAGGTGTGGGGGTCCTTCCACGACCTCCGTGCCGCAGCTAACGCATTAAGCGCCCCGCCTGGGGAGTACGGC
CGCAAGGCTA
>HQ462477
TACGTAGGGTGCAAGCGTTAATCGGAATTACTGGGCGTAAAGCGTGCGCAGGCGGTGATGTAAGACAGTTGTG
AAATCCCCGGGCTCAACCTGGGAACTGCATCTGTGACTGCATCGCTGGAGTGCGGCAGAGGGGGATGGAATTC
CGCGTGTAGCAGTGAAATGCGTAGATATGCGGAGGAACACCGATGGCGAAGGCAATCCCCTGGGCCTGCACTG
ACGCTCATGCACGAAAGCGTGGGGAGCAAACAGGATTAGATACCCTGGTAGTCCACGCCCTAAACGATGTCAA
CTGGTTGTTGGGTCTTCACTGACTCAGTAACGAAGCTAACGCGTGAAGTTGACCGCCTGGGGAGTACGGCCGC
AAGGTTG
>HQ462478
TACGTAGGGGCCTAGCGTTGTCCGGATTTATTGGGCGTAAAGAGCTCGTAGGCGGTTTGGTGAGTCGGGTGTG
AAATCTCCACGCTCAACGTGGAGGGGCCACCCGATACTGCCATGACTAGAGTCCGGTAGGGGAGTGTGGAATT
CCCGGTGTAGCGGTGAAATGCGCAGATATCGGGAGGAACACCAGTAGCGAAGGCGACACTCTGGGCCGGTACT
GACGCTGAGGAGCGAAAGCGTGGGGAGCAAACAGGATTAGATACCCTGGTAGTCCACGCTGTAAACGTTGGGC
ACTAGGTGTGGGACTCTATCGACGGGTTCCGTGCCGTAGCTAACGCATTAAGTGCCCCGCCNGGGGAGTACGG
CCGCAAGGCTA
>HQ462479
TACGAAGGGTGCAAGCGTTACTCGGAATTACTGGGCGTAAAGCGTGCGTAGGTGGTGAGTTAAGTCTGTCATG
AAAGCCCCGGGCTCAACCTGGGAATGGCGATGGATACTGGCTCGCTAGAGTGCGGTAGAGGAGAGTGGAATTC
CCGGTGTAGCAGTGAAATGCGTAGAGATCGGGAGGAACATCAGTTGCGAAGGCGGCTCTCTGGACCAGTACTG
ACACTGAGGCACGAAAGCGTGGGGAGCAAACAGGATTAGATACCCTGGTAGTCCACGCCCTAAACGATGCGAA
CTGGACGTTGGGAGCAATCANGCTCTCAGTGTCGAAGCTNACGCGTTAAGTTCGCCGCCTGGGGAGTACGGTC
GCAAGACTG
>HQ462480
TACGTAGGGGGCAAGCGTTGTCCGGATTTATTGGGCGTAAAGAGCGTGTAGGCGGCCAGATAAGTCTGCTGTG
AAAACTCGAGGCTCAACCTCGAGCTGTCGGCGGAAACTATTTGGCTAGAGTCCGGAAGAGGAAAGTGGAATTC
CTGGTGTAGCGGTGAAATGCGCAGATATCAGGAAGAACACCCGTGGCGAAGGCGGCTTTCTGGGACGGTACTG
ACGCTGAGACGCGAAAGCGTGGGGAGCGAACAGGATTAGATACCCTGGTAGTCCACGCTGTAAACGATGGGTG

CTAGGTGTGGGCGGTGTCGACTCCGTCCGTGCCGAAGCTAACGCATTAAGCACCCCGCCTGGGGAGTACGGCC
GCAAGGCTA
>HQ462481
TACAGAGGGTGCGAGCGTTGTCCGGAATCACTGGGCGTAAAGGGCGCGTAGGTGGCCTGGTAAGTAGGGGGTG
AAATCTCGCGGCTCAACTGCGAGGCTGCCTCCTAAACTGCCGGGCTCGAGCAGAGTAGAGGCAGGTGGAATTC
CCGGTGTAGCGGTGGAATGCGTAGAGATCGGGAAGAACATCGGTGGCGAAGGCGGCCTGCTGGGCTCTTGCTG
ACACTGAGGCGCGACAGCGTGGGGAGCAAACAGGATTAGATACCCTGGTAGTCCACGCCGTAAACGATGGGTA
CTAGGCGCCGGGGGGAGCGACCCCTTCGGTGCCGTCGCTAACGCAATAAGTACCCCGCCTGGGGAGTACGGCC
GCAAGGCTG
>HQ462482
TACGAAGGGGGCTAGCGTTGCTCGGAATCACTGGGCGTAAAGGGTGCGTAGGCGGGTCTTTAAGTCAGGGGTG
AAATCCTGGAGCTCAACTCCAGAACTGCCTTTGATACTGAGGATCTTGAGTTCGGGAGAGGTGAGTGGAACTG
CGAGTGTAGAGGTGAAATTCGTAGATATTCGCAAGAACACCAGTGGCGAAGGCGGCTCACTGGCCCGATACTG
ACGCTGAGGCACGAAAGCGTGGGGAGCAAACAGGATTAGATACCCTGGTAGTCCACGCCGTAAACGATGAATG
CCAGCCGTTAGTGGGTTTACTCACTAGTGGCGCAGCTAACGCTTTAAGCATTCCGCCTGGGGAGTACGGTCGC
AAGATTA
>HQ462483
TACGTAGGGTGCAGGCGTTAATCGGAATTACTGGGCGTAAAGCGTGCGCAGGCGGTTTCTTAAGTCAGATGTG
AAAGCCCCGGGCTTAACCTGGGAACTGCGTTTGAAACTGGGAGACTCGAGTGTGGCAGAGGGGGGTGGAATTC
CACGTGTAGCAGTGAAATGCGTAGATATGTGGAGGAACACCGATGGCGAAGGCAGCCCCCTGGGTTAACACTG
ACGCTCATGCACGAAAGCGTGGGGAGCAAACAGGATTAGATACCCTGGTAGTCCACGCCCTAAACTATGTCAA
CTGGTTGTCGGGGGATTGATTCCCTTGGTAACGAAGCTAACGCGTGAAGTTGACCGCCTGGGGAGTACGGCCG
CAAGGTTA
>HQ462484
TACGTAGGGTGCAAGCGTTAATCGGAATTACTGGGCGTAAAGCGTGCGCAGGCGGTTTTGTAAGTCTGTCGTG
AAATCCCCGGGCTCAACCTGGGAATTGCGATGGAGACTGCAAGGCTAGAATCTGGCAGAGGGGGGTAGAATTC
CACGTGTAGCAGTGAAATGCGTAGAGATGTGGAGGAACACCGATGGCGAAGGCAGCCCCCTGGGTCAAGATTG
ACGCTCATGCACGAAAGCGTGGGGAGCAAACAGGATTAGATACCCTGGTAGTCCACGCCCTAAACGATGTCTA
CTAGTTGTCGGGTTTTAATTAACTTGGTAACGCAGCTAACGCGTGAAGTAGACCGCCTGGGGAGTACGGTCGC
AAGATTA
>HQ462485
TACGTAGGGGGCAAGCGTTGTCCGGATTTATTGGGCGTAAAGAGCTCGTAGGCGGCTTGGCAAGTCGGGTGTG
AAAACTCCAGGCTCAACNTGGAGATGCCACTCGAGACTGCTATGGCTAGAGTCCGGTAGGGGACCACGGAATT
CCTGGTGTAGCGGTGAAATGCGCAGATATCAGGAGGAACACCGGTGGCGAAGGCGGTGGTCTGGGCCGGCACT
GACGCTGAGGAGCGAAAGCGTGGGGAGCAAACAGGATTAGATACCCTGGTAGTCCACGCCGTAAACGTTGGGC
ACTAGGTGTGGGACCTTATCAACGGGTTCCGTGCCGTAGCTAACGCATTAAGTGCCCCGCCTGGGGAGTACGG
CCGCAAGGCTA
>HQ462486
GACGTAGGGGACGAGCGTTGTCCGGATTTATTGGGCGTAAAGCGCGCGTAGGCGGCTCGGACAGTCGGATGTG
AAAATCCGGGGCTCAACCCCGGACCTGCATCCGATACTTCCGGGCTAGAGGTAGGTAGGGGAGATCGGAATTC
CTGGTGTAGCGGTGAAATGCGCAGATATCAGGAGGAACACCGGTGGCGAAGGCGGATCTCTGGGCCTTACCTG
ACGCTGAGGCGCGAAAGCTAGGGGAGCGAACAGGATTAGATACCCTGGTAGTCCTAGCCGTAAACGTTGGGTG
CTAGGTGTGGGGGGACATCGACCCCTTCCGTGCCGAAGCTAACGCATTAAGCACCCCGCCTGGGGAGTACGGC
CGCAAGGCTA
>HQ462487
TACGAAGGGGGCTAGCGTTGTTCGGAATTACTGGGCGTAAAGCGCACGCAGGCGGATTGATAAGTCGGGGGTG
AAATCCCGGGGCTCAACCTCGGAATTGCCTTCGATACTGTCAGTCTTGAGTCCGGGAGAGGTGAGTGGAATTC
CTAGTGTAGAGGTGAAATTCGTAGATATTAGGAAGAACACCAGTGGCGAAGGCGGCTCACTGGCCCGGTACTG
ACGCTCATGTGCGAAAGCGTGGGGAGCAAACAGGATTAGATACCCTGGTAGTCCACGCCGTAAACGATGGATG
CTAGCCGTCGGGGAGCTTGCTCTTCGGTGGCGCAGCTAACGTATTAAGCATCCCGCCTGGGGAGTACGGTCGC
AAGATTA
>HQ462488
TACGAAGGGGGCTAGCGTTGCTCGGAATCACTGGGCGTAAAGCGCGCGTAGGCGGCTTTCTAAGTCAGGGGTG
AAATCCTGGAGCTCAACTCCAGAACTGCCTTTGATACTGGGAAGCTCGAGTCCGGAAGAGGTGAGTGGAACTC

CGAGTGTAGAGGTGAAATTCGTAGATATTCGGAAGAACACCAGTGGCGAAGGCGGCTCACTGGTCCGGTACTG
ACGCTGAGGTGCGAAAGCGTGGGGAGCAAACAGGATTAGATACCCTGGTAGTCCACGCCGTAAACGATGGATG
CTAGCCGTTGGCCGGTTTACCGGTCAGTGGCGNAGCTAACGCATTAAGCATCCCGCCTGGGGAGTACGGTCGC
AAGATTA
>HQ462489
TACGAAGGGGGCTAGCGTTGTTCGGAATTACTGGGCGTAAAGCGCGCGCAGGTGGTCCTTCAAGTCAGGGGTG
AAAGCCCAGAGCTCAACTCTGGAATGGCCTTTGAGACTAGAGGACTTGGGTACGGGAGAGGTGAGTGGAATTC
CGAGTGTAGAGGTGAAATTCGTAGATATTCGGAAGAACACCAGTGGCGAAGGCGGCTCACTGGCCCGTTACCG
ACGCTCAGGCGCGACAGCGTGGGGAGCAAACAGGATTAGATACCCTGGTAGTCCACGCCGTAAACGATGGATG
CTAGCCGTTGGTAAGCATGCTTATCAGTGGCGCAGCTAACGCATTAAGCATCCCGCCTGGGGAGTACGGCCGC
AAGGTTG
>HQ462490
GACAGGGGGCTCGAGCGTTAATCGGAATCACTGGGCTTAAAGGGTGCGTAGGCGGGCTTGTAAGTGTCGTGTG
AAATCCCCCGGCTCAACCGGGGAACTGCGGGGCAAACTGCAAGTCTTGAGGAAGCTAGAGGCCGGTAGAACGA
TTGGTGGAGCGGTGGAATGCGTAGATATCAATCGGAATGCCGAAAGTGAAGACAGCCGGCTGGGGCTTTCCTG
ACGCTGAGGCACGAAAGCGTGGGGAGCAAACGGGATTAGATACCCCGGTAGTCCACGCCCTAAACGATGTGCA
CTAGACTGGAGCGGCTCTGACGTCGTTCTGGTCGTAGAGAAATTGATAAGTGCACCGCCTGGGGAGTACGGTC
GCAAGGCTA
>HQ462491
TACGTAGGGTGCGAGCGTTAATCGGAATTACTGGGCGTAAAGCGTGCGCAGGCGGTTATGCAGGCCAGACGTG
AAAGCCCCGGGCTTAACCTGGGAATGGCGTTTGGGACTGCATGGCTAGAGTACGTCAGAGGGGGGTGGAATTC
CACGTGTAGCAGTGAAATGCGTAGAGATGTGGAGGAACACCGATGGCGAAGGCAGCCCCCTGGGATGATACTG
ACGCTCAGGCACGAAAGCGTGGGGAGCAAACAGGATTAGATACCCTGGTAGTCCACGCCCTAAACGATGTCGA
CTAGTTGTTGGGGAACTTGATTCTTCAGTAACGCAGCTAACGCGTGAAGTCGACCGCCTGGGGAGTACGGTCG
CAAGATTA
>HQ462492
TACGTAGGCAGCGAGCGTTGTTCGGAGTTACTGGGCGTAAAGGGTGTGTAGGCGGTTCTTTAAGTTTGGTGTG
AAATCTCCCGGCTCAACCGGGAGGGTGCGCCGAATACTGAGGGACTAGAGTACGGGAGAGGAAAGTGGAATTC
CTGGTGTAGCGGTGAAATGCGTAGATATCAGGAGGAACACCGGTGGTGTAGACGGCTTTCTGGACCGTAACTG
ACGCTGAGACACGAAAGCGTGGGTAGCAAACAGGATTAGATACCCTGGTAGTCCACGCCCTAAACGATGCATA
TTTGGTGTGGGCAGTTCATTCTGTCCGTGCCGGAGCTAACGCGTTAAATATGCCGCCTGGGGAGTACAGTCGC
AAGGCTG
>HQ462493
TACGAAGGGGGCTAGCGTTGTTCGGAATTACTGGGCGTAAAGCGCACGTAGGCGGATTGTTAAGTCAGAGGTG
AAATCCCGGAGCTCAACTTCGGAACTGCCTTTGATACTGGCAATCTCGAGTCCGGAAGAGGTTAGTGGAATTC
CCAGTGTAGAGGTGAAATTCGTAGATATTGGGAAGAACACCAGTGGCGAAGGCGGCTAACTGGTCCGGTACTG
ACGCTGAGGTGCGAAAGCGTGGGGAGCAAACAGGATTAGATACCCTGGTAGTCCACGCCGTAAACTATGGGTG
CTAGCCGTTGGGAAGCTTGCTTTTCAGTGGCGCAGCTAACGCATTAAGCACCCCGCCTGGGGAGTACGGTCGC
AAGATTA
>HQ462494
TACGGAGGGTGCTAGCGTTGTTCGGAATCACTGGGCGTAAAGGGCGTGTAGGCGGTCTGTTAAGTCTGATGTG
AAATCCCCTCGCTCAACGAGGGAACTGCGTCGGATACTGGCAGACTCGAGTACCGGAGAGGAAGGTGGAATTC
CCGGTGTAGCGGTGAAATGCGTAGATATCGGGAGGAACACCGGTGGCGAAGGCGGCCTTCTGGACGGATACTG
ACGCTGAGACGCGAAAGCGTGGGGAGCAAACAGGATTAGATACCCTGGTAGTCCACGCTGTAAACGATGGGCA
CTAGGTGTCGCGGGTATTGACCCCTGCGGTGCCGCAGCTAACGCATTAAGTGCCCCGCCTGGGGACTACGGCC
GCAAGGTTA
>HQ462495
TACGGGGGGAGCAAGCGTTGTTCGGATTTACTGGGCGTAAAGGGCGCGTAGGCGGCCAGCGCAAGTCAGTTGT
GAAATCTCCGGGCTTAACTCGGAAAGGTCAACTGATACTGCGCGGCTAGAGTGCAGAAGGGGCAACTGGAATT
CTCGGTGTAGCGGTGAAATGCGTAGATATCGAGAGGAACACCTGCGGCGAAGGCGGGTTGCTGGGCTGACACT
GACGCTGAGGCGCGAAAGCCAGGGGAGCGAACGGGATTAGATACCCCGGTAGTCCTGGCCTTAAACGATGGAT
GCTTGGTGTCTGGGGTTTTATAGTCCCCGGGTGCCGCAGCTAACGCGTTAAGCATCCCGCCTGGGGAGTACGG
TCGCAAGACTG
>HQ462496

TACGAGGGGTGCAAACGTTATTCGGATTTATTGGGCGTAAAGGGTGCGTAGACGGCATACTAAGTTCGTTGTT
AAATCTTCCGGCTCAACCGGAAGCTCGCAGTGAAAACTAGTAAGCTTGAGGATGGAAGAGAGAAGTGGAATTC
CTGGAGTAGCGGTAAAATGCGTAGATCTCAAGAGGAACACCGATGGCGAAGGCAGCTTCTTGGTCCATTCCTG
ACGTTGATGCACGAAAGCGTGGGGAGCAAACAGGATTAGATACCCTGGTAGTCCACGCTGTAAACGATGATTA
CTGGATGTCAGACTCGCTCTGCGAGGCTGGTATCGAAGCTAACGCGTTAAGTAATCCGCCTGGGAACTACGAT
CGCAAGATTA
>HQ462497
TACAGAGGGGGCCAGCGTTATTCGGAATTATTGGGCGTAAAGGGCGCGTAGGCGGCCATGCGTGTCTTCCGTG
AAATCCCTCGGCTTAACTGAGGACGTGCGGAAGAAACTGCAAGGCTTGAGTCCGGGAGAGGGGGGTGGAATTC
CCAGTGTAGCGGTGAAATGCGTAGATATTGGGAGGAACACCGGTGGCGAAGGCGGCCCCCTGGACCGGTACTG
ACGCTGAGGCGCGAAAGCGTGGGTAGCAAACGGGATTAGATACCCCGGTAGTCCACGCCGTAAACGATGGGCA
CTTGGTGTCGCGGGTATCGACCCCTGCGGTGCCGAAGCTAACGCATTAAGTGCCCCGCCTGGGGAGTACGGTC
GCAAGGCTG
>HQ462498
GACAGAGGGGGCAAGCGTTGCTCGGAATTACTGGGCGTAAAGGGCGCGTAGGCGGGATGGTAAGTCGGTCGTG
AAATCCCTCGGCTCAACCGAGGAACGTCGATCGATACTACCGTTCTTGAGGATTGCAGAGGAGACTGGAATTC
CCGGTGTAGCGGTGAAATGTGTAGAGATCGGGAGGAACACCGGTGGCGAAGGCGGGTCTCTGGGCAATTCCTG
ACGCTGAGGCGCGAAAGCGTGGGGAGCAAACGGGATTAGATACCCCGGTAGTCCACGCTGTAAACGATGAGCA
CTAGGTGTGGGGGGGGTTCAGTCCCTTCCGTGCCGCAGCTAACGCATTAAGTGCTCCGCCTGGGGGAGTACGG
CCGCAAGGTTG
>HQ462499
TACGAAGGGGGCTAGCGTTGCTCGGAATTACTGGGCGTAAAGGGAGCGTAGGTGGACAGTTAAGTCAGTGGTG
AAAGCCCAGGGCTCAACCCTGGAATTGCCTTTGATACTGATTGTCTTGAGTATGGGAGAGGTATGCGGAACTC
CGAGTGTAGAGGTGAAATTCGTAGATATTCGGAAGAACACCAGTGGCGAAGGCGGCATACTGGCCCATTACTG
ACGCTGAGGCTCGAAAGCGTGGGGAGCAAACAGGATTAGATACCCTGGTAGTCCACGCTGTAAACGATGAGTG
CTAGTTGTCGGCATGCATGCATGTCGGTGACGCAGCTAACGCATTAAGCACTCCGCCTGGGGAGTACGGTCGC
AAGATTA
>HQ462500
CACGTAGGGGGCAAGCGTTGTCCGGATTTATTGGGCGTAAAGAGCTCGTAGGCGGTTCGGTAAGTCGGGTGTG
AAACCTCCAGGCTCAACCTGGAGACGCCACCTGATACTGCTGTGACTCGAGTCCGGTAGGGGAGCGTGGAATT
CCTGGTGTAGCGGTGAAATGCGCAGATATCAGGAGGAACACCAGCGGCGAAGGCGGCGCTCTGGGCCGGAACT
GACGCTGAGGAGCGAAAGCGTGGGTAGCAAACAGGATTAGATACCCTGGTAGTCCACGCCGTAAACGTTGGGC
ACTAGGTGTGGGGTCTTATCAACGGATTCCGTGCCGTAGCTAACGCATTAAGTGCCCCGCCTGGGGAGTACGG
CCGCAAGGCTA
>HQ462501
TACGAAGGGGGCTAGCGTTGTTCGGAATTACTGGGCGTAAAGCGCACGTAGGCGGATTGTTAAGTCAGAGGTG
AAATCCCGGAGCTCAACTTCGGAACTGCCTTTGATACTGGCAATCTCGAGTCCGGAAGAGGTTAGTGGAATTC
CCAGTGTAGAGGTGAAATTCGTAGATATTGGGAAGAACACCAGTGGCGAAGGCGGCTAACTGGTCCGGTACTG
ACGCTGAGGTGCGAAAGCGTGGGGAGCAAACAGGATTAGATACCCTGGTAGTCCACGCCGTAAACTATGGGTG
CTAGCCGTTGGGAAGCTTGCTTTTCAGTGGCGCAGCTAACGCATTAAGCACCCCGCCTGGGGAGTACGGTCGC
AAGATTA
>HQ462502
GACAGAGGTCCCGAGCGTTGTTCGGATTCATTGGGCGTAAAGGGTGTGTAGGAGGTTGGGTAAGTCAGGTGTG
AAATCTCAGAGCTTAACTCTGAAACTGCGCTTGATACTGCTCAGCTAGAGGATCGGAGGGGGTAACGGAATTT
ATGGTGTAGCAGTGAAATGCGTAGATATCATAAGGAACACCGGTGGCGAAGGCGGTTACCTGGAAGATTCCTG
ACTCTGAAACACGAAAGCCAGGGGAGCAAACGGGATTAGATACCCCGGTAGTCCTGGCCGTAAACGGTGTGCA
TTGGGCGTANGCGGATTCGACCCCGTCTGTGTCGTAGATAACTCATTAAATGCACCGCCTGGGAAGTACGGTC
GCAAGATTA
>HQ462503
TACGTAGGGTGCAAGCGTTAATCGGAATTACTGGGCGTAAAGCGTGCGCAGGCGGCTTTGCAAGACAGATGTG
AAATCCCCGGGCTTAACCTGGGAACTGCATTTGTGACTGCATGGCTAGAGTGCGGCAGAGGGGGATGGAATTC
CGCGTGTAGCAGTGAAATGCGTAGATATGCGGAGGAACACCGATGGCGAAGGCAATCCCCTGGGCCTGCACTG
ACGCTCATGCACGAAAGCGTGGGGAGCAAACAGGATTAGATACCCTGGTAGTCCACGCCCTAAACGATGTCGA

CTGGTTGTTGGACGGCTTGCTGTTCAGTAACGAAGCTAACGCGTGAAGTTGACCGCCTGGGGAGTACGGCCGC
AAGGTTG
>HQ462504
TACGTAGGGACCAAGCGTTGTTCGGATTTACTGGGCGTAAAGGGCGCGTAGGCGGCTTGACAAGTCACTTGTG
AAATCTCCGGGCTTAACTCGGAACGGCCAAGTGAAACTGTCATGCTAGAGTGCAGAAGGGGCAATCGGAATTC
TTGGTGTAGCGGTGAAATGCGTAGATATCAAGAGGAACACCTGAGGTGAAGACGGGTTGCTGGGCTGACACTG
ACGCTGAGGCGCGAAAGCCAGGGGAGCAAACGGGATTAGATACCCCGGTAGTCCTGGCCCTAAACGATGAATA
CTTGGTGTCTGGAGTTATTAGTGCTCCGGGTGCCGTCGCTAACGTTTTTAGTATTCCGCCTGGGGAGTACGCT
CGCAAGAGTG
>HQ462505
TACGGAGGGTGCAAGCGTTATCCGGATTCACTGGGTTTAAAGGGTGCGTAGGTGGGCAGGTAAGTCAGTGGTG
AAATCTTTGGGCTTAACCCGAAAACTGCCATTGATACTATTTGTCTTGAATATTGTGGAGGTCAGCGGAATAT
GTCATGTAGCGGTGAAATGCTTAGATATGACATAGAACACCAATTGCGAAGGCAGCTGGCTACACATATATTG
ACACTGATGCACGAAAGCGTGGGGATCAAACAGGATTAGATACCCTGGTAGTCCACGCCCTAAACGATGGATA
CTCGACATACGCGATATACTGTGTGTGTCTGAGCGAAAGCATTAAGTATCCCACCTGGGAAGTACGATCGCAA
GATTG
>HQ462506
GACAGAGGTCCCGAGCGTTGTTCGGATTCATTGGGCGTAAAGGGTGTGTAGGAGGTTGGGTAAGTCAGGTGTG
AAATCTCAGAGCTTAACTCTGAAACTGCGCTTGATACTGCTCAGCTAGAGGATCGGAGGGGGTAACGGAATTT
ATGGTGTAGCAGTGAAATGCGTAGATATCATAAGGAACACCGGTGGCGAAGGCGGTTACCTGGAAGATTCCTG
ACTCTGAAACACGAAAGCCAGGGGAGCAAACGGGATTAGATACCCCGGTAGTCCTGGCCGTAAACGGTGTGCA
TTGGGCGTAGGCGGATTCGACCCCGTCTGTGTCGTAGATAACTCATTAAATGCACCGCCTGGGAAGTACGGTC
GCAAGATTA
>HQ462507
TACGTAGGGTGCGAGCGTTAATCGGAATTACTGGGCGTAAAGCGTGCGTAGGCGGTTCGTTAAGTTCGTCGTG
AAAGCCCCGGGCTCAACCTGGGAATGGCGATGGAAGCTGGCGGGCTTGAGTATGGCAGAGGGGGGTGGAATTC
CGCGTGTAGCAGTGAAATGCGTAGAGATGCGGAGGAACACCGATGGCGAAGGCAGCCCCCTGGGCCAATACTG
ACGCTCAGGCACGAAAGCGTGGGGAGCAAACAGGATTAGATACCCTGGTAGTCCACGCCCTAAACGATGCTCA
CTAGGTGTTGGGGAAGCGATTCCTTGGTACCGTAGCTAACGCGTGAAGTGAGCCGCCTGGGGAGTACGGCCGC
AAGGTTA
>HQ462508
TACAGAGGCCACAAGCATTAGTCGGAATCACTGGGCTTAAAGGGTGCGTAGGTGGCTCACCAAGTGCTTTGTG
AAATCCTACGGCTCAACCGTGGAACAGCAGAGCATACTGGTGGGCTTGAGGCAAGTAGGGGCTGACGGAACAG
TAGGTGGAGCGGTGAAATGCGTAGATATCTACTGGAACGCCGATGGTGAAGACGGTCAGCTGGGCTTGTCCTG
ACACTGAGGCACGAAAGCGTGGGGAGCAAACAGGATTAGATACCCTGGTAGTCCACGCTGTAAACGATGCACA
CTGGACCGGTGCGGTTTTGACGCCGTATCGGTCGTAGTAAAAACGATAAGTGTGCCGCCTGGGGAGTACGGTC
GCAAGATTA
>HQ462509
TACGAAGGGGGCTAGCGTTGCTCGGAATCACTGGGCGTAAAGCGCACGTAGGCGGGTCGCTGAGTCGGGGGTG
AAATCCTGGAGCTTAACTCCAGAACTGCCTTCGATACTGGCGATCTTCGAGTCCGGGAGAGGTGGGTGGAACT
GCGAGTGTAGAGGTGAAATTCGTAGATATTCGCAAGAACACCAGTGGCGAAGGCGGCTCACTGGCCCGGTACT
GACGCTGAGGTGCGAAAGCGTGGGGAGCAAACAGGATTAGATACCCTGGTAGTCCACGCCGTAAACGATGGAT
GCTAGCCGTTGGCAGGCTTGCCTGTCAGTGGCGCAGCTAACGCATTAAGCATCCCGCCTGGGGAGTACGGTCG
CAAGATTA
>HQ462510
TACAGAGGTCCCAAGCGTTGTTCGGATTCATTGGGCGTAAAGGGTGTGTAGGTGGCGCCGTAAGTCGGGTGTG
AAATCTCGGGGCTTAACTCCGAAACTGCATTCGATACTGCGGTGCTTGAGGACTGGAGAGGAGACTGGAATTC
ATGGTGTAGCAGTGAAATGCGTAGAGATCATGAGGAAGACCAGTGGCGAAGGCGGGTCTCTGGACAGTTCCTG
ACACTGAGACACGAAGGCCAGGGGAGCAAACGGGATTAGATACCCCGGTAGTCCTGGCAGTAAACGGTGCACG
TTTGGTGTGGGGGGATTCGACCCCTTCTGCGCCGGAGCTAACGCGTTAAACGTGCCGCCTGGGGAGTACGGTC
GCAAGATTA
>HQ462511
TACGTAGGTGGCAAGCGTTGTCCGGATTTATTGGGCGTAAAGAGCGCGCAGGCGGTTCGTTAAGTCCGAGGTG
AAATCTCCCGGCTCAACTGGGAGGGGTCTTCGGATACTGGCGGACTTGAGGAGTGCAGAGGTGAGTGGAATTC

TCGGTGTAGTGGTGAAATGCGTAGATATCGAGAGGAACACCAGCGGCGAAGGCGGCTCACTGGGCACCACCTG
ACGCTGAGGCGCGAAAGCGTGGGGAGCGAACCGGATTAGATACCCGGGTAGTCCACGCCGTAAACGATGGGCA
CTAGGTGTGAGGGGTATTGACCCCCTTCGTGCCGAAGCTAACGCAATAAGTGCCCCGCCTGGGGAGTACGGCC
GCAAGGCTA
>HQ462512
TACGGGGGGAGCAAGCGTTGTTCGGATTTACTGGGCGTAAAGGGCGCGTAGGCGGCCACCGCAAGTCGATTGT
GAAATCTCCGGGCTTAACCCGGAAAGGTCAACCGATACTGCGGGGCTAGAGTGCGGAAGGGGCAACTGGAATT
CTTGGTGTAGCGGTGAAATGCGTAGATATCAAGAGGAACACCTGCGGCGAAGGCGGGTTGCTGGGCCGACACT
GACGCTGATGCGCGAAAGCTAGGGGAGCGAACGGGATTAGATACCCCGGTAGTCCTAGCCTTAAACGATGAAT
GCTTGGTGTCTGGGGTTTTATAGTCCCCGGGTGCCGTCGCTAACGCTTTAAGCATTCCGCCTGGGGAGTACGG
TCGCAAGACTG
>HQ462513
TACAGAGGGTGCGAGCGTTGTCCGGAATCACTGGGCGTAAAGGGCGTGTAGGTGGCTTGATAAGCCGCGTGTG
AAATCCCGGGGCTCAACCCCGGAACGGCACGCGGGACTGTCAGGCTAGAGCATGGTAGAGGCGAGTGGAATTC
CTGGTGTAGCGGTGGAATGCGTAGAGATCAGGAAGAACACCGGTGGCGAAGGCGGCTCGCTGGGCCATTGCTG
ACACTGAGGCGCGAAAGCGTGGGGAGCAAACAGGATTAGATACCCTGGTAGTCCACGCTGTAAACGATGGGCA
CTAGGCGTCGGGGGGAGCGACCCTCCCGGTGCCGTCGCTAACGCAATAAGTGCCCCGCCTGGGGAGTACGGCC
GCAAGGTTG
>HQ462514
TACGTAGGTGGCAAGCGTTGTCCGGATTTATTGGGCGTAAAGGATGCGTAGGCGGACATTTAAGTCAGATGTG
AAATACCCGAGCTTAACTTGGGTGCTGCATTTGAAACTGGGTGTCTAGAGTGCAGGAGAGGTAAGTGGAATTC
CTAGTGTAGCGGTGAAATGCGTAGAGATTAGGAAGAACACCAGTGGCGAAGGCGACTTACTGGACTGTAACTG
ACGCTGAGGCATGAAAGCGTGGGGAGCAAACAGGATTAGATACCCTGGTAGTCCACGCCGTAAACGATGAATA
CTAGGTGTCGGGGGTCGAACCTCGGTGCCGCCGTTAACACATTAAGTATTCCGCCTGGGGAGTACGATCGCAA
GATTA
>HQ462515
TACGGAGGGTGCGAGCGTTGTTCGGAATTATTGGGCGTAAAGCGCGTGTAGGCGGCTCCTTAAGTCTGATGTG
AAAGCCCTGGGCTCAACCCGGGAAGTGCATTGGAAACTGGGGAACTTGAATACGGGAGAGGGTAGTGGAATTC
CTGGTGTAGGAGTGAAATCCGTAGATATCAGGAGGAACACCGGTGGCGAAGGCGGCTACCTGGACCGATATTG
ACGCTGAGACGCGAAAGCGTGGGGAGCAAACAGGATTAGATACCCTGGTAGTCCACGCCGTAAACGATGAGTA
CTAGGTGTTGCGGGTATTGACCCCTGCAGTGCCGCAGCTAACGCATTAAGTACTCCGCCTGGGAAGTACGGTC
GCAAGACTA
>HQ462516
TACGTAGGGGGCAAGCGTTGTCCGGAATCATTGGGCGTAAAGAGCGTGTAGGCGGCCCGATAAGTCCGCTGTG
AAAGTCCAGGGCTCAACCCTGGGATGCCGGTGGATACTGTCGGGCTCGAGTCCGGAAGAGGAGTGTGGAATTC
CTGGTGTAGCGGTGAAATGCGCAGATATCAGGAGGAACACCAATGGCGAAGGCAGCACTCTGGGACGTGACTG
ACGCTGAGACGCGAAAGCGTGGGGAGCAAACAGGATTAGATACCCTGGTAGTCCACGCTGTAAACGATGGGCA
CTAGNNGTGGGGGGNGTCGACTCCCTCCGTGCCGAAGCTAACGCATTAAGTGCCCCGCCTGGGGAGTACGGCC
GCAAGGCTA
>HQ462517
TACGAAGGGGGCTAGCGTTGCTCGGAATCACTGGGCGTAAAGGGTGCGTAGGCGGGTCTTTAAGTCAGGGGTG
AAATCCTGGAGCTCAACTCCAGAACTGCCTTTGATACTGAGGATCTTGANTTCGGGAGAGGTGAGTGGAACTG
CGAGTGTAGAGGTGAAATTCGTAGATATTCGCAAGAACACCAGTGGCGAAGGCGGCTCACTGGCCCGATACTG
ACGCTGAGGCACGAAAGCGTGGGGAGCAAACAGGATTAGATACCCTGGTAGTCCACGCCGTAAACGATGAATG
CCAGCCGTTAGTGGGTTTACTCACTAGTGGCGCAGCTAACGCTTTAAGCATTCCGCCTGGGGAGTACGGTCGC
AAGATTA
>HQ462518
TACGAAGGGGGCTAGCGTTGCTCGGAATCACTGGGCGTAAAGCGCACGTAGGCGGATCTTTAAGTCAGGGGTG
AAATCCTGGAGCTCAACTCCAGAACTGCCTTTGATACTGGGGATCTCGAGTCCGGAAGAGGTGAGTGGAACTC
CGAGTGTAGAGGTGAAATTCGTAGATATTCGGAAGAACACCAGTGGCGAAGGCGGCTCACTGGTCCGGTACTG
ACGCTGAGGTGCGAAAGCGTGGGGAGCAAACAGGATTAGATACCCTGGTAGTCCACGCCGTAAACGATGGATG
CTAGCCGTTGGCCGGTTTACCGGTCAGTGGCGCAGCTAACGCATTAAGCATCCCGCCTGGGGAGTACGGTCGC
AAGATTA
>HQ462519

TACGTAGGGTGCGAGCGTTAATCGGAATTACTGGGCGTAAAGCGTGCGCAGGCGGTTCTCCAAGTCCGTTGTG
AAAGCCCCGGGCTCAACCTGGGAATGGCAGTGGAAACTAGGGGACTTGAATCTGGCAGAGGGGGGTGGAATTC
CACGTGTAGCAGTGAAATGCGTAGAGATGTGGAGGAACACCGATGGCGAAGGCAGCCCCCTGGGCTGAGATTG
ACGCTCAGGCACGAAAGCGTGGGGAGCAAACAGGATTAGATACCCTGGTAGTCCACGCCCTAAACGATGTCAA
CTAGGTGTTGGGGAAGCGATTTCTCAGTACCGCAGCTAACGCGTGAAGTTGACCGCCTGGGGAGTACGGTCGC
AAGATTA
>HQ462520
CACGTAGGGGGCAAGCGTTGTCCGGATTCATTGGGCGTAAAGAGCTCGTAGGCGGTTCGATAAGTCGGGTGTG
AAACCTCCAGGCTCAACCTGGAGACGCCACCCGATACTGTTGTGACTAGAGTCCAGTAGGGGAGCGTGGAATT
CCTGGTGTAGCGGTGAAATGCGCAGATATCAGGAGGAACACCAGCGGCGAAGGCGGCGCTCTGGGCTGGAACT
GACGCTGAGGAGCGAAAGCGTGGGGAGCGAACAGGATTAGATACCCTGGTAGTCCACGCCGTAAACGTTGGGT
ACTAGGTGTGGGGTCTTATCAACGGATTCCGTGCCGAAGCTAACGCATTAAGTACCCCGCCTGGGGAGTACGG
CCGCAAGGCTA
>HQ462521
TACGTAGGGGCCTAGCGTTGTCCGGATTTATTGGGCGTAAAGAGCTCGTAGGCGGTTTGGTGAGTCGGGTGTG
AAATCTCCACGCTCAACGTGGAGGGGCCACCCGATACTGCCATGACTAGAGTCCGGTAGGGGAGTGTGGAATT
CCCGGTGTAGCGGTGAAATGCGCAGATATCGGGAGGAACACCAGTAGCGAAGGCGACACTCTGGGCCGGTACT
GACGCTGAGGAGCGAAAGCGTGGGGAGCAAACAGGATTAGATACCCTGGTAGTCCACGCTGTAAACGTTGGGC
ACTAGGTGTGGGACTCTATCGACGGGTTCCGTGCCGTAGCTAACGCATTAAGTGCCCCGCCTGGGGAGTACGG
CCGCAAGGCTA
>HQ462522
GACAGAGGGTGCGAACGTTGTTCGGAATTACTGGGCGTAAAGCGCGTGTAGGCTGCTCGGAAAGTCGGGTGTG
AAATCCCTGGGCTCAACCCAGGAAGTGCACTCGAAACTTCCAAGCTCGAGTCCTGGAGAGGAAAGCGGAATTC
TCGGTGTAGAGGTGAAATTCGTAGATATCGAGAGGAACACCGGTGGCGAAGGCGGCTTTCTGGACAGTGACTG
ACGCTGAGACGCGAAAGCGTGGGGAGCAAACAGGATTAGATACCCTGGTAGTCCACGCCGTAAACGATGGGTG
CTAGGTGTCGCANGCTTTGACCCCTGCGGTGCCGTAGCTAACGCATTAAGCACCCCGCCTGGGGAGTACGGCC
GCAAGGCTA
>HQ462523
TACGTAGGGTGCAAGCGTTGTCCGGAATTATTGGGCGTAAAGAGCTCGTAGGCGGTTTGTCGCGTCGAATGTG
AAAACCCGGGGCTCAACTCCGGGCCTGCATTCGATACGGGCAGACTAGAGTTCGGTAGGGGAGACTGGAATTC
CTGGTGTAGCGGTGAAATGCGCAGATATCAGGAGGAACACCGGTGGCGAAGGCGGGTCTCTGGGCCGATACTG
ACGCTGAGGAGCGAAAGCGTGGGGAGCAAACAGGATTAGATACCCTGGTAGTCCACGCCGTAAACGTTGGGCG
CTAGGTGTGGGGGTCCTTCCACGACCTCCGTGCCGCAGCTAACGCATTAAGCGCCCCGCCTGGGGAGTACGGC
CGCAAGGCTA
>HQ462524
TACGTAGGGTGCGAGCGTTAATCGGAATTACTGGGCGTAAAGCGTGCGCAGGCGGTTCTGCAAGTCAGCTGTG
AAAGCCCCGGGCTTAACCTGGGAATGGCGGTTGAAACTGCAGGACTAGAATCTGGCAGAGGGGGGTGGAATTC
CACGTGTAGCAGTGAAATGCGTAGAGATGTGGAGGAACACCGATGGCGAAGGCAGCCCCCTGGGCTGAGATTG
ACGCTCAGGCACGAAAGCGTGGGGAGCAAACAGGATTAGATACCCTGGTAGTCCACGCCCTAAACGATGCCAA
CTAGGTGTCGGGAGCGCAAGTTCTTGGTACCGCAGCTAACGCGTGAAGTTGACCGCCTGGGGAGTACGGTCGC
AAGATTA
>HQ462525
GACGTAGGGCGCGAGCGTTGTCCGGATTTATTGGGCGTAAAGAGCTCGTAGGCGGCTTGTCGCGTCGACTGTG
AAATCCCGCGGCTCGACCGCGGGTCTGCAGTCGATACGGGCAGGCTAGAGTTCGGTAGGGGAGACTGGAATTC
CTGGTGTAGCGGTGAAATGCGCAGATATCAGGAGGAACACCGATGGCGAAGGCAGGTCTCTGGGCCGATACTG
ACGCTGAGGAGCGAAAGCGTGGGGAGCGAACAGGATTAGATACCCTGGTAGTCCACGCTGTAAACGTTGGGCG
CTAGGTGTGGGGGACCTCTCCGGTTCTCTGTGCCGCAGCTAACGCATTAAGCGCCCCGCCTGGGGAGTACGGC
CGCAAGGCTA
>HQ462526
TACGAAGGGGGCTAGCGTTGTTCGGAATTACTGGGCGTAAAGCGTATGCAGGCGGATCGTTAAGTCAGGGGTG
AAATCCCGAGGCTCAACCTCGGAACTGCCTTTGATACTGACGATCTTGAGTCCGGGAGAGGTGAGTGGAACTG
CGAGTGTAGAGGTGAAATTCGTAGATATTCGCAAGAACACCAGTGGCGAAGGCGGCTCACTGGCCCGGTACTG
ACGCTGAGATACGAAAGCGTGGGGAGCAAACAGGATTAGATACCCTGGTAGTCCACGCCGTAAACGATGAATG

CTAGCCGTCGGTGGGCATGCCCTTCGGTGGCGCAGCTAACGCAATAAGCATTCCGCCTGGGGAGTACGATCGC
AAGATTA
>HQ462527
TACGGGGGGGGCAAGCGTTGTTCGGAATTACTGGGCGTAAAGGGCTCGTAGGCGGCCAACTAAGTCAAACGTG
AAATCCCTCGGCTTAACCGGGGAACTGCGCTTGATACTGGATGGCTTGAGATTGGGAGAGGGATGCGGAATTC
CAGGTGTAGCGGTGAAATGCGTAGATATCTGGAGGAACACCGGTGGCGAAGGCGGCATCCTGGACCAACACTG
ACGCTGAGGAGCGAAAGCCAGGGGAGCAAACGGGATTAGATACCCCGGTAGTCCTGGCCCTAAACGATGAATG
CTTGGTGTGGCGGGTATCGATCCCTGCCGTGCCGAAGCTAACGCATTAAGCATTCCGCCTGGGGAGTACGGTC
GCAAGGCTG
>HQ462528
TACAGAGGTGGCAAGCGTTGTTCGGAATTACTGGGCGTAAAGGGCGCGTAGGCGGTCCTACAAGTCCCGCGTG
AAAGCCCCCGGCTCAACTGGGGAATGGCGCGGGAAACTGTAGGGCTTGAGTTCGGGAGAGGGAAGCGGAATTC
CGGGTGTAGCGGTGAAATGCGTAGATATCCGGAGGAACACCGGTGGCGAAGGCGGCTTCCTGGACCGACACTG
ACGCTGAGGCGCGAAAGCTAGGGGAGCAAACGGGATTAGATACCCCGGTAGTCCTAGCTGTAAACGATGAGTG
CTGGGTGTAGGGGGTATCAACCCCCCCTGTGCCGAAGCTAACGCATTAAGCACTCCGCCTGGGGAGTACGGTC
GCAAGGCTG
>HQ462529
TACGTAGGGGGCTAGCGTTGTCCGGAATCATTGGGCGTAAAGCGCGCGTAGGCGGCCCGGTAAGTCCGCTGTG
AAAGTCAAAGGCTCAACCTTTGAATGTCAGCGGATACTGCCGGGCTAGAGTCCGGAAGAGGCGAGTGGAACTC
CTGGTGTAGCGGTGGAATGCGCAGATATCAGGAAGAACACCAACGGCGAAGGCAGCTCGCTGGGACGGAACTG
ACGCTAAGGCGCGAAAGCGTGGGGAGCGAACAGGATTAGATACCCTGGTAGTCCACGCCGTAAACGATGGGCA
CTAGGTGTGGGAGGTGTCGACTCCTCCCGTGCCGGCGCTAACGCATTAAGTGCCCCGCCTGGGGAGTACGGCC
GCAAGGCTA
>HQ462530
TACGTAGGGTGCGAGCGTTGTCCGGAATTATTGGGCGTAAAGAGCTCGTAGGCGGTGTGTCGCGTCGGCCGTG
AAAACCTGCAGCTTAACTGTGGGCTTGCGGTCGATACGGGCATCACTGGAGTTCGGCAGGGGAGACTGGAATT
CCTGGTGTAGCGGTGAAATGCGCAGATATCAGGAGGAACACCGGTGGCGAAGGCGGGTCTCTGGGCCGATACT
GACGCTGAGGAGCGAAAGCGTGGGGAGCGAACAGGATTAGATACCCTGGTAGTCCACGCCGTAAACGGTGGGC
GCTAGGTGTGGGGGCCATTCCACGGTCTCTGTGCCGTAGCTAACGCATTAAGCGCCCCGCCTGGGGAGTACGG
CCGCAAGGCTA
>HQ462531
TACGGGGGGAGCAAGCGTTGTTCGGATTTACTGGGCGTAAAGGGCGCGTAGGCGGTCAGTGCAAGTCAGTTGT
GAAATCTCCGGGCTTAACTCGGAAAGGTCAACTGATACTGCTCGACTAGAGTGCGGAAGGGGCAACTGGAATT
CTTGGTGTAGCGGTGAAATGCGTAGATATCAAGAGGAACACCTGCGGCGAAGGCGGGTTGCTGGGCCGACACT
GACGCTGAGGCGCGAAAGCTAGGGGAGCGAACGGGATTAGATACCCCGGTAGTCCTAGCCTTAAACGATGAAT
GCTTGGTGTCTGGGGTTATAAAGTCCCCGGGTGCCGTCGCTAACGCTTTAAGCATTCCGCCTGGGGAGTACGG
TCGCAAGACTG
>HQ462532
TACGAAGGGGGCTAGCGTTGTTCGGAATTACTGGGCGTAAAGCGCACGTAGGCGGATTGTTAAGTCAGAGGTG
AAATCCCGGAGCTCAACTTCGGAACTGCCTTTGATACTGGCAATCTAGAGTCCGGAAGAGGTTAGTGGAATTC
CCAGTGTAGAGGTGAAATTCGTAGGTATTGGGAAGAACACCAGTGGCGAAGGCGGCTAACTGGTCCGGTACTG
ACGCTGAGGTGCGAAAGCGTGGGGAGCAAACAGGATTAGATACCCTGGTAGTCCACGCCGTAAACTATGGGTG
CTAGCCGTCAGGAAGCTTGCTTTTTGGTGGCGCAGCTAACGCATTAAGCACCCCGCCTGGGGAGTACGGTCGC
AAGATTA
>HQ462533
TACGTAGGGGGCAAGCGTTGTTCGGAATTACTGGGCGTAAAGGGCGCGTAGGCGGCACGGCAAGTCAGGAGTG
AAAGGCCTGGGCTCAACCGAGGAACTGCTTTTGAGACTGCCGAGCTAGAGTGCGGAAGGGGCAACTGGAATTC
CCAGTGTAGCGGTGAAATGCGTAGATATTGGGAAGAACACCTGAGGCGAAGGCGGGTTGCTGGGCCGACACTG
ACGCTGAGGCGCGAAAGCCAGGGGAGCAAACGGGATTAGATACCCCGGTAGTCCTGGCCCTAAACGATGGATA
CTTGGTGTATGGGGTTCTTCGAGTCCCCGTGTGCCGGAGCTAACGCGTTAAGTATCCCGCCTAGGGAGTACGG
TCGCAAGGCTG
>HQ462534
TACGAAGGGGGCTAGCGTTGTTCGGAATTACTGGGCGTAAAGCGTATGCAGGCGGATCGTTAAGTCGGGGGTG
AAATCCCGAGGCTCAACCTCGGAACTGCCTTCGATACTGGCGATCTTCGAGTCCGGGAGAGGTGAGTGGAACT

CCGAGTGTAGAGGTGAAATTCGTAGATATTCGGAAGAACACCAGTGGCGAAGGCGGCTCACTGGCNCGGTACT
GACGCTGAGATACGAAAGCGTGGGGAGCAAACAGGATTAGATACCCTGGTAGTCCACGCTGTAAACGATGGGT
GCTAGCCGTTGGGCAGCATGCTGTTCGGTGGCGCAGCTAACGCATTAAGCACCCCGCCTGGGGAGTACGATCG
CAAGATTA
>HQ462535
TACGTAGGTGACAAGCGTTGTCCGGATTTACTGGGCGTAAAGAGCGCGCAGGCGGTCGTTCAAGTCGAGTGTG
AAAGCCCCCGGCTCAACTGGGGAGGGTCACTCGATACTGATCGACTCGAAGGCAGGAGAGGGAAGTGGAATTC
CCGGTGTAGTGGTGAAATGCGTAGATATCGGGAGGAACACCAGTGGCGAAGGCGACTTCCTGGCCTGTTCTTG
ACGCTGAGGCGCGAAAGCTAGGGGAGCAAACGGGATTAGATACCCCGGTAGTCCTAGCCGTAAACGATGGACA
CTAGGTGTTGGTGGTATCAACCCCGCCAGTGCCGAAGCTAACGCATTAAGTGTCCCGCCTGGGGAGTACGGCC
GCAAGGCTA
>HQ462536
TACGGAGGATCCAAGCGTTATCCGGATTTATTGGGTTTAAANGGGTGCGTAGGCGGGTTTGTAAGTCAGTGGT
GAAATCCTGCAGCTTAACTGTAGAACTGCCATTGATACTGCAAGTCTTGAGTACATATGATGTGGGCGGAATG
TGTAGTGTAGCGGTGAAATGCTTAGATATTACACAGAACACCGATTGCGAAGGCAGCTCACAAAACTGTAACT
GACGCTGAGGCACGAAAGCGTGGGGAGTGAACAGGATTAGATACCCTGGTAATCCACGCCTTAAACGATGATT
ACTCGATGTGTGCGATACACAGCACGCGTCCTAGCGAAAGCGTTAAGTAATCCACCTGGGGAGTACGATCGCA
AGGTTG
>HQ462537
TACGTAGGAGACAAGCGTTATCCGGATTCATTGGGCGTAAAGTGTGCGTAGGCTCCTTGAAAAGTCGTACCTT
AAATACTAAGGCTTAACCTTGGGACTGGGTACGATACTTTTAAGGTAGAGGATTGTCAGGGGTACTGGAACAG
TTAATGTAGCAGTGAAATGCGTTGATATTAACTGGAACACCAAAGGCGAAGGCAAGTACCTGGGATTTTCCTG
ACGCTGAGGCACGAAAGCTAGGGGAGCGAAACAGATTAGATACCTGTGTAGTCCTAGCCGTAAACGATGTCTG
CTAGCTTTTTCCGACTTGTCGGAAGAGGCGCAAGCTAACGCGTTAAGCAGACCGCCTGGGGAGTACGGCCGCA
AGGCTA
>HQ462538
TACGAAGGGGGCTAGCGTTGCTCGGAATCACTGGGCGTAAAGCGCACGTAGGCGGGTCGCTGAGTCGGGGGTG
AAATCCTGGAGCTTAACTCCAGAACTGCCTTCGATACTGGCGATCTTCGAGTCCGGGAGAGGTGGGTGGAACT
GCGAGTGTAGAGGTGAAATTCGTAGATATTCGCAAGAACACCAGTGGCGAAGGCGGCTCACTGGCCCGGTACT
GACGCTGAGGTGCGAAAGCGTGGGGAGCAAACAGGATTAGATACCCTGGTAGTCCACGCCGTAAACGATGGAT
GCTAGCCGTTGGCAGGCTTGCCTGTCAGTGGCGCAGCTAACGCATTAAGCATCCCGCCTGGGGAGTACGGTCG
CAAGATTA
>HQ462539
TACGTAGGGTGCAAGCGTNGTCCGGAATTATTGGGCGTAAAGAGCTTGTAGGCGGTTTGTCGCGTCTGCTGTG
AAATTTCGGGGCTCAACCCCGAACTTGCAGTGGGTACGGGCAGACTAGAGTGTGGTAGGGGAGACTGGAATTC
CTGGTGTAGCGGTGAAATGCGCAGATATCAGGAGGAACACCGATGGCGAAGGNAGGTCTCTGGGCCACTACTG
ACGCTGAGAAGCGAAAGCATGGGGAGCGAACAGGATTAGATACCCTGGTAGTCCGCGCCGTAAACGTTGGGAA
CTAGGTGTGGGTCTCATTCCACGAGGTCCGTGCCGCAGCTAACGCATTAAGTTCCCCGCCTGGGGAGTACNGC
CGCAANGCTA
>HQ462540
TACGTAGGGTGCAAGCGTTGTCCGGAATTATTGGGCGTAAAGAGCTTGTAGGCGGTTTGTCGCGTCTGCTGTG
AAATTTCGGGGCTCAACCCCGAACTTGCAGTGGGTACGGGCAGACTAGAGTGTGGTAGGGGAGACTGGAATTC
CTGGTGTAGCGGTGAAATGCGCAGATATCAGGAGGAACACCGATGGCGAAGGCAGGTCTCTGGGCCACTACTG
ACGCTGAGAAGCGAAAGCATGGGGAGCGAACAGGATTAGATACCCTGGTAGTCCGCGCCGTAAACGTTGGGAA
CTAGGTGTGGGTCTCATTCCACGAGGTCCGTGCCGCAGCTAACGCATTAAGTTCCCCGCCTGGGGAGTACGGC
CGCAAGGCTA
>HQ462541
TACGTAGGGGGCAAGCGTTGTCCGGATTTATTGGGCGTAAAGAGCTCGTAGGCGGTTCAGTAAGTCACGTGTG
AAATCTCCGCGCTCAACGCGGAGGCGCCACGTGATACTGCTGTGACTCGAGTCCGGTAGGGGAGTGTGGAATT
CCCGGTGTAGCGGTGAAATGCGCAGAGATCGGGAGGAACACCCGTAGCGAAGGCGACACTCTGGGCCGGTACT
GACGCTGAGGAGCGAAAGCGTGGGGAGCAAACAGGATTAGATACCCTGGTAGTCCACGCCGTAAACGTTGGGC
ACTAGGTGTGGGGATCTATCGACGGTTTCCGTGCCGCAGCTAACGCATTAAGTGCCCCGCCTGGGGAGTACGG
CCGCAAGGCTA
>HQ462542

TACGAAGGGGGCTAGCGTTGTTCGGATTTACTGGGCGTAAAGCGCACGTAGGCGGATTGTTAAGTCAGGGGTG
AAATCCCGGAGCTCAACTCCGGAACTGCCTTTGATACTGGCAATCTTGAGGCTGGAAGAGGTTAGTGGAATTC
CCAGTGTAGAGGTGAAATTCGTAGATATTGGGAAGAACACCAGTGGCGAAGGCGGCTAACTGGTCCAGATCTG
ACGCTGAGGTGCGAAAGCGTGGGGAGCAAACAGGATTAGATACCCTGGTAGTCCACGCCGTAAACTATGGGTG
CTAGCTGTCAGCGGGCTTGCTCGTTGGTGGCGCAGCTAACGCATTAAGCACCCCGCCTGGGGAGTACGGTCGC
AAGATTA
>HQ462543
TACGAAGGGGGCTAGCGTTGCTCGGAATCACTGGGCGTAAAGCGCACGTAGGCGGGTCGCTGAGTCGGGGGTG
AAATCCTGGAGCTTAACTCCAGAACTGCCTTCGATACTGGCGATCTTCGAGTCCGGGAGAGGTGGGTGGAACT
GCGAGTGTAGAGGTGAAATTCGTAGATATTCGCAAGAACACCAGTGGCGAAGGCGGCTCACTGGCCCGGTACT
GACGCTGAGGTGCGAAAGCGTGGGGAGCAAACAGGATTAGATACCCTGGTAGTCCACGCCGTAAACGATGGAT
GCTAGCCGTTGGCAGGCTTGCCTGTCAGTGGCGCAGCTAACGCATTAAGCATCCCGCCTGGGGAGTACGGTCG
CAAGATTA
>HQ462544
TACGGGGGGNGCAAGCGTTGTTCGGATTTANTGGGCGTAAAGGGCGCGTAGGCGGCCTCCGCAAGTCAATTGT
GAAATCTCCGGGCTTAACTCGGAAAGGTCAACTGATACTGCGGGGCTAGAGTGCGGAAGGGGCAACTGGAATT
CTCGGTGTAGCGGTGAAATGCGTAGATATCGAGAGGAACACCTGCGGCGAAGGCGGGTTGCTGGGCCGACACT
GACGCTGAGGCGCGAAAGCTAGGGGAGCGAACGGGATTAGATACCCCGGTAGTCCTAGCCTTAAACGATGAAT
GCTTGGTGTCTGGGGTTATATAGTCCCCGGGTGCCGTCGCTAACGCTTTAAGCATTCCGCCTGGGGAGTACGG
TCGCAAGACTG
>HQ462545
TACGGGGGGAGCAAGCGTTGTTCGGATTTACTGGGCGTAAAGGGCGCGTAGGCGGCCAGCGCAAGTCAGTTGT
GAAATCTCCGGGCTTAACTCGGAAAGGTCAACTGATACTGCGCGGCTAGAGTGCGGAAGGGGCAACTGGAATT
CTCGGTGTAGCGGTGAAATGCGTAGATATCGAGAGGAACACCTGCGGCGAAGGCGGGTTGCTGGGCCGACACT
GACGCTGAGGCGCGAAAGCCAGGGGAGCGAACGGGATTAGATACCCCGGTAGTCCTGGCCTTAAACGATGGAT
GCTTGGTGTCTGGGGTTTTATAGTCCCCGGGTGCCGCAGCTAACGCGTTAAGCATCCCGCCTGGGGAGTACGG
TCGCAAGACTG
>HQ462546
TACGTAGGGTGCGAGCGTTAATCGGAATTACTGGGCGTAAAGCGTGCGCAGGCGGTGATGTAAGACAGATGTG
AAATCCCCGGGCTCAACCTGGGAACTGCATTTGTGACTGCATCGCTAGAGTACGGTAGAGGGGGATGGAATTC
CGCGTGTAGCAGTGAAATGCGTAGATATGCGGAGGAACACCGATGGCGAAGGCAATCCCCTGGACCTGTACTG
ACGCTCATGCACGAAAGCGTGGGGAGCAAACAGGATTAGATACCCTGGTAGTCCACGCCCTAAACGATGTCAA
CTGGTTGTTGGGTCTTCACTGACTCAGTAACGAAGCTAACGCGTGAAGTTGACCGCCTGGGGAGTACGGCCGC
AAGGTTG
>HQ462547
TACGTAGGGTGCAAGCGTTAATCGGAATTACTGGGCGTAAAGCGTGCGCAGGCGGCTTTGCAAGACAGATGTG
AAATCCCCGGGCTTAACCTGGGAACTGCATTTGTGACTGCATGGCTCGAGTGCGGCAGAGGGGGATGGAATTC
CGCGTGTAGCAGTGAAATGCGTAGATATGCGGAGGAACACCGATGGCGAAGGCAATCCCCTGGGCCTGCACTG
ACGCTCATGCACGAAAGCGTGGGGAGCAAACAGGATTAGATACCCTGGTAGTCCACGCCCTAAACGATGTCAA
CTGGTTGTTGGACGGCTTGCTGTTCAGTAACGAAGCTAACGCGTGAAGTTGACCGCCTGGGGAGTACGGCCGC
AAGGTTG
>HQ462548
TACGAAGGGGGCTAGCGTTGCTCGGAATCACTGGGCGTAAAGCGCACGTAGGCGGGTTCTTAAGTCAGGGGTG
AAATCCTGGAGCTCAACTCCAGAACTGCCCTTGATACTGAGAATCTTGAGTCCGGGAGAGGTGAGTGGAACTG
CGAGTGTAGAGGTGAAATTCGTAGATATTCGCAAGAACACCAGTGGCGAAGGCGGCTCACTGGCCCGGTACTG
ACGCTGAGGTGCGAAAGCGTGGGGAGCAAACAGGATTAGATACCCTGGTAGTCCACGCTGTAAACGATGGATG
CTAGCCGTTGGGGAGCTTGCTCTTCAGTGGCGCAGCTAACGCATTAAGCATCCCGCCTGGGGAGTACGGTCGC
AAGATTA
>HQ462549
TACAGAGGTCTCNAGCGTTGTTCGGATTCATTGGGCGTAAAGGGTGCGTAGGTGGCGCCGTAAGTGGGGTGTG
AAATTTCGGAGCTTAACTCCGAAACTGCATTCCATACTGCGGTGCTTGAGGACTGGAGAGGAGACTGGAATTT
ACGGTGTAGCAGTGAAATGCGTAGAGATCGTAAGGAAGACCAGTGGCGAAGGCGGGTCTCTGGACAGTTCCTG
ACACTGAGGCACGAAGGCCAGGGGAGCAAACGGGATTAGATACCCCGGTAGTCCTGGCAGTAAACGGTGCACG

TTTGGTGTGAGAGGATTCGACCCCTTTCGCGCCGGAGCTAACGCGTTAAACGTGCCGCCTGGGGAGTACGGTC
GCAAGATTA
>HQ462550
TACGTAGGGTGCAAGCGTTAATCGGAATTACTGGGCGTAAAGCGTGCGCAGGCGGCTTTGCAAGACAGATGTG
AAATCCCCGGGCTTAACCTGGGAACTGCATTTGTGACTGCATGGCTGGAGTGCGGCAGAGGGGGATGGAATTC
CGCGTGTAGCAGTGAAATGCGTAGATATGCGGAGGAACACCGATGGCGAAGGCAATCCCCTGGGCCTGCACTG
ACGCTCATGCACGAAAGCGTGGGGAGCAAACAGGATTAGATACCCTGGTAGTCCACGCCCTAAACGATGTCAA
CTGGTTGTTGGACGGCTTGCTGTTCAGTAACGAAGCTAACGCGTGAAGTTGACCGCCTGGGGAGTACGGCCGC
AAGGTTG
>HQ462551
TACGTAGGGTGCGAGCGTTGTCCGGAATTATTGGGCGTAAAGAGCTCGTAGGCGGTGTGTCGCGTCGGCCGTG
AAAACCTGCAGCTTAACTGTGGGCGTGCGGTCGATACGGGCATCACTGGAGTTCGGCAGGGGAGACTGGAATT
CCTGGTGTAGCGGTGAAATGCGCAGATATCAGGAGGAACACCGGTGGCGAAGGCGGGTCTCTGGGCCGATACT
GACGCTGAGGAGCGAAAGCGTGGGGAGCAAACAGGATTAGATACCCTGGTAGTCCACGCCGTAAACGGTGGGC
GCTAGGTGTGGGGGCCATTCCACGGTCTCTGTGCCGCAGCTAACGCATTAAGCGCCCCGCCTGGGGAGTACGG
CCGCAAGGCTA
>HQ462552
TACGTAGGGGGCTAGCGTTGTCCGGAATCATTGGGCGTAAAGCGCGTGTAGGCGGTCCGGTAAGTCCGCTGTG
AAAGTCGGGGGCTCAACCCTCGAATGCCGGTGGATACTGTCGGGCTAGAGTGCGGAAGAGGCGAGTGGAATTC
CTGGTGTAGCGGTGAAATGCGCAGATATCAGGAGGAACACCAATTGCGAAGGCAGCTCGCTGGGACGTTACTG
ACGCTGAGACGCGAAAGCGTGGGGAGCAAACAGGATTAGATACCCTGGTAGTCCACGCTGTAAACGATGGGCA
CTAGGTGNGGGGGGNGTCGACTCCCTCCGTGCCGAAGCTAACGCATTAAGTGCCCCGCCTGGGGAGTACGGCC
GCAAGGCTA
>HQ462553
TACGGAGGATCCAAGCGTTATCCGGATTTATTGGGTTTAAAGGGTGCGTAGGCGGGTTTGTAAGTCAGTGGTG
AAATCCTGCAGCTTAACTGTAGAACTGCCATTGATACTGCAAGTCTTGAGTACATATGATGTGGGCGGAATGT
GTAGTGTAGCGGTGAAATGCTTAGATATTACACAGAACACCGATTGCGAAGGCAGCTCACAAAACTGTAACTG
ACGCTGAGGCACGAAAGCGTGGGGAGTGAACAGGATTAGATACCCTGGTAATCCACGCCTTAAACGATGATTA
CTCGATGTGTGCGATACACAGCACGCGTCCTAGCGAAAGCGTTAAGTAATCCACCTGGGGAGTACGATCGCAA
GGTTG
>HQ462554
TACGTAGGGTGCAAGCGTTAATCGGAATTACTGGGCGTAAAGCGTGCGCAGGCGGTTATGTAAGACAGATGTG
AAATGCCCGGGCTTAACCTGGGAACTGCATTTGTGACTGCATGGCTAGAATCTGGCAGAGGGGGGTAGAATTC
CACGTGTAGCAGTGAAATGCGTAGAGATGTGGAGGAACACCGATGGCGAAGGCAGCCCCCTGGGTCAAGATTG
ACGCTCATGCACGAAAGCGTGGGNAGCAAACAGGATTAGATACCCTGGTAGTCCACGCCCTAAACGATGTCTA
CTAGTTGTCGGGTTTTAATTAACTTGGTAACGCAGCTAACGCGTGAAGTAGACCGCCTGGGGAGTACGGTCGC
AAGATTA
>HQ462555
CACGTAGGGGGCAAGCGTTGTCCGGATTCATTGGGCGTAAAGAGCTCGTAGGCGGTTCGATAAGTCGGGTGTG
AAACCTCCAGGCTCAACCTGGAGACGCCACTCGATACTGTCGTGACTAGAGTCCAGTAGGGGAGCGCGGAATT
CCTGGTGTAGCGGTGAAATGCGCAGATATCAGGAGGAACACCAGCGGCGAAGGCGGCGCTCTGGGCTGGAACT
GACGCTGAGGAGCGAAAGCGTGGGGAGCGAACAGGATTAGATACCCTGGTAGTCCACGCCGTAAACGTTGGGT
ACTAGGTGTGGGGTCTTATCAACGGATTCCGTGCCGCAGCTAACGCATTAAGTACCCCGCCTGGGGAGTACGG
CCGCAAGGCTA
>HQ462556
TACAGAGGTGGCAAGCGTTGTTCGGAATTACTGGGCGTAAAGGGCGCGTAGGCGGTCCCGTAAGTTCCGCGTG
AAATCCCTCGGCTCAACCGGGGAATGGCGCGGAAAACTGCGGGGCTTGAGTTCGGGAGAGGGAAGCGGAATTC
CGGGTGTAGCGGTGAAATGCGTAGATATCCGGAGGAACACCGGTGGCGAAGGCGGCTTCCTGGACCGACACTG
ACGCTGAGGCGCGAAAGCTAGGGGAGCAAACGGGATTAGATACCCCGGTAGTCCTAGCTGTAAACGATGAGTG
CTGGGTGTAGGGGGTATCAACCCCCCCTGTGCCGAAGCTAACGCATTAAGCACTCCGCCTGGGGAGTACGGTC
GCAAGGCTG
>HQ462557
TACGAAGGGGGCTAGCGTTGTTCGGAATTACTGGGCGTAAAGCGCGCGCAGGCGGCTTTTCAAGTCAGGGGTG
AAAGCCCGGAGCTCAACTCCGGAACTGCCTTTGAAACTGTGAAGCTTGAGTACGGGAGAGGTGAGTGGAATTC

CCAGTGTAGAGGTGAAATTCGTAGATATTGGGAAGAACACCGGTGGCGAAGGCGGCTCACTGGCCCGTTACTG
ACGCTCAGGCGCGACAGCGTGGGGATCAAACAGGATTAGATACCCTGGTAGTCCACGCCGTAAACGATGAACG
CTAGCCGTTGGGCAGCTTGCTGTTCAGTGGCGCAGCTAACGCATTAAGCGTTCCGNCTGGGGAGTACGGCCGC
AAGGTTG
>HQ462558
TACGGGGGGAGCANGCGTTGTTCGGATTTACTGGGCGTAAAGGGCGCGTAGGCGGTCAGCACAAGTCAGTTGT
GAANTCTCCGAGCTTAACTCGGAACGGTCAACTGATACTGTGCGACTAGAGTGCGGAAGGGGCAACTGGAATT
CTTGGTGTAGCGGTGAAATGCGTAGATATCAAGAGGAACACCTGCGGCGAAGGCGGGTTGCTGGGCCGACACT
GACGCTGAGGCGCGAAAGCCAGGGGAGCGAACGGGATTAGATACCCCGGTAGTCCTGGCCTTAAACGATGAAT
GCTTGGTGTCTGGGGTTATAAAGTCCCCGGGTGCCGTCGCTAACGCTTTAAGCATTCCGCCTGGGGAGTACGG
TCGCAAGACTG
>HQ462559
CACGTAGGCACCAAGCGTTGTCCGGATTTATTGGGCGTAAAGAGCTCGTAGGCGGTTCAGTAAGTCGGGTGTG
AAAACTTTGGGCTTAACCCAAAGCGTGCATCTGATACCGCTGTGACTTGAGTTCGGTAGGGGAGTGGGGAATT
CCTAGTGTAGCGGTGAAATGCGCAGATATTAGGAGGAACACCGGTGGCGAAGGCGCCACTCTGGGCCGAAACT
GACGCTGAGGAGCGAAAGCATGGGTAGCAAACAGGATTAGATACCCTGGTAGTCCATGCCGTAAACGTTGGGC
ACTAGGTGTGGGTTCCAACCAACGGAATCCGCGCCGTCGCTAACGCATTAAGTGCCCCGCCTGGGGAGTACGG
CCGCAAGGTTA
>HQ462560
TACAGAGACCTCAAGCGTTATCCGGAATCATTGGGCGTAAAGCGTGCCGATAGGTGGTTTTGTTAGTCTTGGG
TAAAAGCCAGAAGCTTAACTTCTGAGTTTGCTTAAGATACTGCAAAACTAGAGGGGCAAAGAGGTGCTTGGAA
CGAACGGTGTAGTAGTGAAATGCGTTGATATCGTTCGGAACACCGAAAGCGAAGGCAGAGCACTGGGTGCCAC
CTGACACTGCTAGGACGAAAGCGTGGGTAGCGAATGGGATTAGATACCCCAGTAGTCCACGCTGTAAACGATG
AATACTAGGCATGCGCGAGTATCGACCCTCGTGCGAGCCGTAGCTAACGCGTTAAGTATTCCGCCTGGGGAGT
ACGGTCGCAAGACTA
>GDUPB3C07IVPKT
TACAGAGGGGGCAAGCGTTATTCGGAATTATTGGGCGTAAAGGGCGCGTAGGCGGCCTTGCAAGTGGAAGGTG
AAATCCCTCGGCTTAACCGAGGAACTGCCTTCCAGACTGCTGGGCTTGAGACCGGGAGAGGTGAGTGGAATTC
CCAGTGTAGCGGTGAAATGCGTAGATATTGGGAGGAACACCAGTGGCGAAGGCGGCTCACTGGACCGGTACTG
ACGCTGATGCGCGAAAGCGTGGGGAGCAAACGGGATTAGATACCCCGGTAGTCCACGCCGTAAACGATGAGTG
CTTGGTGTAGCGGGTATCGACCCCTGCTGTGCCGAAGTCAACACATTAAGCACTCCGCCTGGGGAGTACGGTC
GCAAGGCTG
>GDUPB3C07IWQYP
TACGAAGGGGGCTAGTGTTGTTCGGAATTACTGGGCGTAAAGGGTATGTAGGCGGATCGTTAAGTCGGGGGTG
AAATCCCGAGGCTCAACCTCGGAACTGCCTTCGATACTGGCGACCTTCGAGTCCGGGAGAGGTGAGTGGAACT
CCGAGTGTAGAGGTGAAATTCGTAGATATTCGGAAGAACACCAGTGGCGAAGGCGGCTCACTGGCCCGGTACT
GACGCTGAAATACGAAAGCGTGGGGAGCAAACAGGATTAGATACCCTGGTAGTCCACGCTGTAAACGATGGGT
GCTAGCCGTTGGGCAGCATGCTGTTCGGTGGCGCAGCTAACGCATTAAGCACCCCGCCTGGGGAGTACGATCG
CAAGATTA
