# Supplementary figures and images for: FlowClus: efficiently filtering and denoising pyrosequenced amplicons
Source: BMC Bioinformatics. 2015 Mar 27;16(1):105. doi: 10.1186/s12859-015-0532-1 (PMC4380255; doi:10.1186/s12859-015-0532-1)

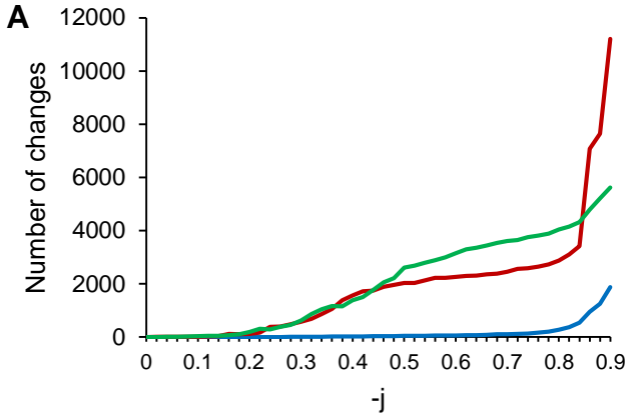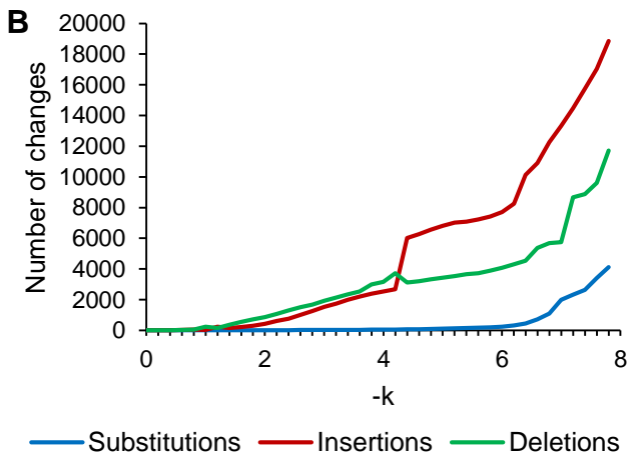

Supplement: Additional file 4: — Effects of altering the parameters of FlowClus when using a trie. The numbers of changes to the reads made during denoising with a trie was determined. A: Effects of changing the constant denoising value. B: Effects of using different multiples for the distances based on the standard deviations of Balzer et al. [22]. [file 12859_2015_532_MOESM4_ESM.pdf]

**A**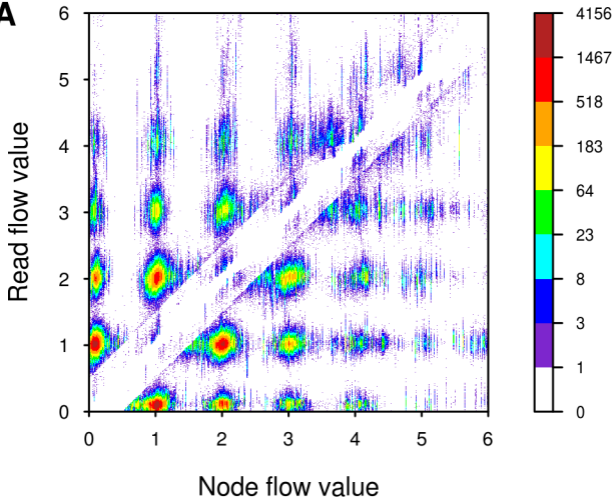**B**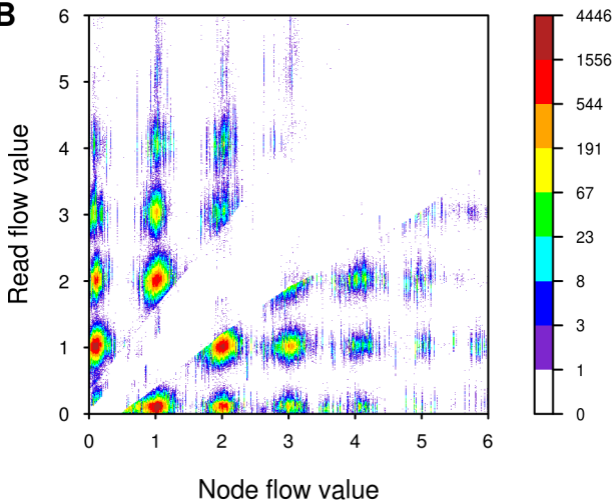

Supplement: Additional file 5: — Denoising “misses” with FlowClus when using a trie. Levelplots of the flow values that were judged as being distinct, based on the following user-selected parameters. A: A constant value of 0.50. B: A multiple of five distances based on the standard deviations of Balzer et al. [22]. [file 12859_2015_532_MOESM5_ESM.pdf]

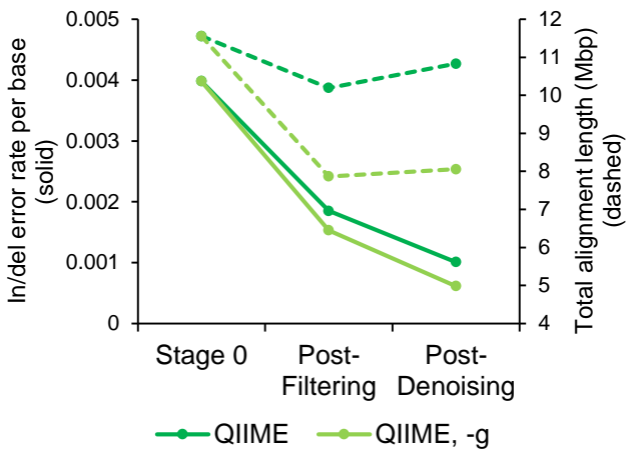

Supplement: Additional file 7: — Analyzing a mock community dataset by the QIIME denoising pipeline. A comparison of the error rates (solid lines) and total sequence alignment length (dashed lines) of the Titanium mock community dataset [6] analyzed by the QIIME denoising pipeline, with and without the -g filtering option. [file 12859_2015_532_MOESM7_ESM.pdf]

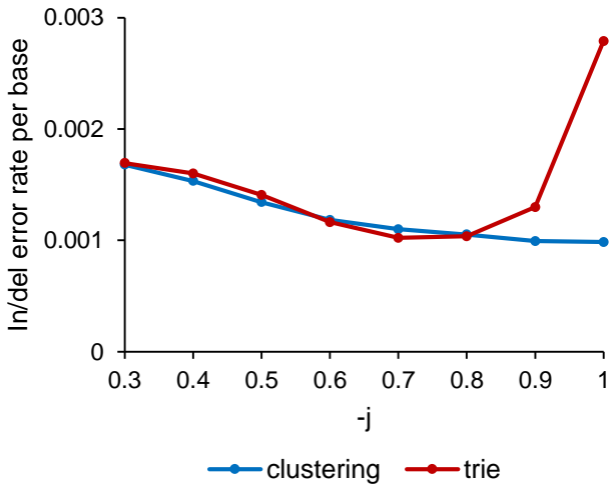

Supplement: Additional file 8: — Comparisons of the error rates when denoising by clustering vs. by trie at different denoising distances. [file 12859_2015_532_MOESM8_ESM.pdf]
