# Supplementary material for: FlowClus: efficiently filtering and denoising pyrosequenced amplicons
Source: BMC Bioinformatics. 2015 Mar 27;16(1):105. doi: 10.1186/s12859-015-0532-1 (PMC4380255; doi:10.1186/s12859-015-0532-1)
Supplement: Additional file 6: — Comparisons of the run-times (in seconds) of different denoising pipelines. A: Titanium mock community dataset of Quince et al. [6]. B: Baseline dataset of Krych et al. [25]. C: Combined dataset (baseline, synbiotic, and placebo) of Krych et al. [25]. [file 12859_2015_532_MOESM6_ESM.pdf]

**Comparisons of the run-times (in seconds) of different denoising algorithms**

A. Titanium mock community dataset of Quince *et al.* [6].

| | FlowClus | AmpliconNoise | QIIME |
|---|---|---|---|
| Number of reads denoised | 29,387 | 25,438 | 29,036 |
| Filtering | 6 | 84 | 195 |
| Denoising | clustering: 15<br>trie: 2 | 34,067 | 23,882 |

B. Baseline dataset of Krych *et al.* [25].

| | FlowClus | AmpliconNoise | QIIME |
|---|---|---|---|
| Number of reads denoised | 528,788 | 422,150 | 525,183 |
| Filtering | 121 | 2,877 | 3,670 |
| Denoising | clustering: 6,432<br>trie: 36 | 226,256* | 679,478* |

* Run in parallel over 16 cores. Actual CPU times: AmpliconNoise 3,321,403 sec; QIIME 10,823,468 sec.

C. Combined dataset (baseline, synbiotic, and placebo) of Krych *et al.* [25].

| | FlowClus |
|---|---|
| Number of reads denoised | 1,479,465 |
| Filtering | 332 |
| Denoising | clustering: 41,187<br>trie: 97 |
